# COVID-19 and Mental Illnesses in Vaccinated and Unvaccinated People

**DOI:** 10.1001/jamapsychiatry.2024.2339

**Published:** 2024-08-21

**Authors:** Venexia M. Walker, Praveetha Patalay, Jose Ignacio Cuitun Coronado, Rachel Denholm, Harriet Forbes, Jean Stafford, Bettina Moltrecht, Tom Palmer, Alex Walker, Ellen J. Thompson, Kurt Taylor, Genevieve Cezard, Elsie M. F. Horne, Yinghui Wei, Marwa Al Arab, Rochelle Knight, Louis Fisher, Jon Massey, Simon Davy, Amir Mehrkar, Seb Bacon, Ben Goldacre, Angela Wood, Nishi Chaturvedi, John Macleod, Ann John, Jonathan A. C. Sterne

**Affiliations:** 1 Population Health Sciences, University of Bristol, Bristol, United Kingdom; 2 Medical Research Council Integrative Epidemiology Unit, University of Bristol, Bristol, United Kingdom; 3 Department of Surgery, University of Pennsylvania Perelman School of Medicine, Philadelphia; 4 Medical Research Council Unit for Lifelong Health and Ageing, University College London, London, United Kingdom; 5 Centre for Longitudinal Studies, University College London, London, United Kingdom; 6 National Institute for Health and Care Research, Bristol Biomedical Research Centre, Bristol, United Kingdom; 7 Health Data Research UK South-West, Bristol, United Kingdom; 8 Faculty of Epidemiology and Population Health, London School of Hygiene & Tropical Medicine, London, United Kingdom; 9 The Bennett Institute for Applied Data Science, Nuffield Department of Primary Care Health Sciences, University of Oxford, Oxford, United Kingdom; 10 Department of Twin Research and Genetic Epidemiology, School of Life Course & Population Sciences, Faculty of Life Sciences & Medicine, King’s College London, London, United Kingdom; 11 School of Psychology, University of Sussex, Falmer, United Kingdom; 12 British Heart Foundation Cardiovascular Epidemiology Unit, Department of Public Health and Primary Care, University of Cambridge, Cambridge, United Kingdom; 13 Victor Phillip Dahdaleh Heart and Lung Research Institute, University of Cambridge, Cambridge, United Kingdom; 14 Centre for Mathematical Sciences, School of Engineering, Computing and Mathematics, University of Plymouth, Plymouth, United Kingdom; 15 The National Institute for Health and Care Research Applied Research Collaboration West at University Hospitals Bristol and Weston, United Kingdom; 16 British Heart Foundation Centre of Research Excellence, University of Cambridge, Cambridge, United Kingdom; 17 National Institute for Health and Care Research Blood and Transplant Research Unit in Donor Health and Behaviour, University of Cambridge, Cambridge, United Kingdom; 18 Health Data Research UK Cambridge, Wellcome Genome Campus and University of Cambridge, Cambridge, United Kingdom; 19 Cambridge Centre of Artificial Intelligence in Medicine, Cambridge, United Kingdom; 20 Swansea University Medical School, University of Swansea, Swansea, United Kingdom

## Abstract

**Importance:**

Associations have been found between COVID-19 and subsequent mental illness in both hospital- and population-based studies. However, evidence regarding which mental illnesses are associated with COVID-19 by vaccination status in these populations is limited.

**Objective:**

To determine which mental illnesses are associated with diagnosed COVID-19 by vaccination status in both hospitalized patients and the general population.

**Design, Setting, and Participants:**

This study was conducted in 3 cohorts, 1 before vaccine availability followed during the wild-type/Alpha variant eras (January 2020-June 2021) and 2 (vaccinated and unvaccinated) during the Delta variant era (June-December 2021). With National Health Service England approval, OpenSAFELY-TPP was used to access linked data from 24 million people registered with general practices in England using TPP SystmOne. People registered with a GP in England for at least 6 months and alive with known age between 18 and 110 years, sex, deprivation index information, and region at baseline were included. People were excluded if they had COVID-19 before baseline. Data were analyzed from July 2022 to June 2024.

**Exposure:**

Confirmed COVID-19 diagnosis recorded in primary care secondary care, testing data, or the death registry.

**Main Outcomes and Measures:**

Adjusted hazard ratios (aHRs) comparing the incidence of mental illnesses after diagnosis of COVID-19 with the incidence before or without COVID-19 for depression, serious mental illness, general anxiety, posttraumatic stress disorder, eating disorders, addiction, self-harm, and suicide.

**Results:**

The largest cohort, the pre–vaccine availability cohort, included 18 648 606 people (9 363 710 [50.2%] female and 9 284 896 [49.8%] male) with a median (IQR) age of 49 (34-64) years. The vaccinated cohort included 14 035 286 individuals (7 308 556 [52.1%] female and 6 726 730 [47.9%] male) with a median (IQR) age of 53 (38-67) years. The unvaccinated cohort included 3 242 215 individuals (1 363 401 [42.1%] female and 1 878 814 [57.9%] male) with a median (IQR) age of 35 (27-46) years. Incidence of most outcomes was elevated during weeks 1 through 4 after COVID-19 diagnosis, compared with before or without COVID-19, in each cohort. Incidence of mental illnesses was lower in the vaccinated cohort compared with the pre–vaccine availability and unvaccinated cohorts: aHRs for depression and serious mental illness during weeks 1 through 4 after COVID-19 were 1.93 (95% CI, 1.88-1.98) and 1.49 (95% CI, 1.41-1.57) in the pre–vaccine availability cohort and 1.79 (95% CI, 1.68-1.90) and 1.45 (95% CI, 1.27-1.65) in the unvaccinated cohort compared with 1.16 (95% CI, 1.12-1.20) and 0.91 (95% CI, 0.85-0.98) in the vaccinated cohort. Elevation in incidence was higher and persisted longer after hospitalization for COVID-19.

**Conclusions and Relevance:**

In this study, incidence of mental illnesses was elevated for up to a year following severe COVID-19 in unvaccinated people. These findings suggest that vaccination may mitigate the adverse effects of COVID-19 on mental health.

## Key Points

**Question:**

What are the associations between mental illnesses and diagnosed COVID-19 by vaccination status in patients hospitalized for COVID-19 and the general population?

**Findings:**

In this cohort study, depression, serious mental illness, general anxiety, posttraumatic stress disorder, eating disorders, addiction, self-harm, and suicide were elevated during weeks 1 through 4 after COVID-19 diagnosis compared with before or without COVID-19. Incidence was lower in people who were vaccinated when they had COVID-19 and incidence was higher, and persisted longer, after hospitalization for COVID-19.

**Meaning:**

The findings support recommendation of COVID-19 vaccination in the general population and particularly among those with mental illness, who may be at higher risk of both SARS-CoV-2 infection and adverse outcomes following COVID-19.

## Teaser

This cohort study evaluates associations between mental illness and COVID-19 by vaccination status.

## Introduction

SARS-CoV-2 infection, and consequent COVID-19, are associated with subsequent mental illnesses in both hospital- and population-based studies,^1,2^ including both common mental health difficulties, such as anxiety and depressive symptoms,^3^ and serious mental illness, including psychotic disorders.^4^ Potential mechanisms include physiological pathways, such as inflammation and microvascular changes, and psychosocial effects, such as anxiety about the potential outcomes of COVID-19, including post–COVID-19 condition. Previous studies have identified associations of COVID-19 with mental illnesses in both hospitalized patients^5,6^ and the general population.^1,7,8^ Differentiating between hospitalized patients and the general population may provide insights into the implications of COVID-19 severity for subsequent mental illnesses.^9^

Rapid rollout of COVID-19 vaccination was a crucial component of the public health response. Although the impacts of vaccination in preventing and reducing the severity of COVID-19 are well established,^10,11^ there is limited evidence regarding the implications of vaccination for other adverse outcomes of COVID-19, including mental illnesses. We did not identify any studies investigating differences in mental illnesses following COVID-19 by vaccination status. Furthermore, rates of SARS-CoV-2 infection, vaccination, and disease severity may differ by sociodemographic and health factors,^12,13,14,15,16^ so mental health outcomes may also vary between subgroups.

Using electronic health record data from more than 18 million people, we examined associations of diagnosed COVID-19 with subsequent mental illnesses prior to vaccine availability and for unvaccinated and vaccinated people after vaccination became available. We compared rates of mental illnesses after COVID-19 with rates before or without COVID-19. We also examined associations in subgroups defined by COVID-19 severity, age, sex, ethnicity, prior mental illness, and prior COVID-19. Ethnicity data were reported because disparities in COVID-19 outcomes by ethnic group have been reported.^17^ Follow-up of those diagnosed with COVID-19 during the first year of the pandemic was for up to 2 years postdiagnosis.

## Methods

### Study Design and Data Sources

Our study used OpenSAFELY-TPP, which provides secure, privacy-protecting access to linked data from 24 million people registered with general practices (GPs) in England using TPP SystmOne software. These data include primary care data linked via pseudonymized National Health Service number to the Secondary Uses Service secondary care data, the Office of National Statistics Death Registry, the Second Generation Surveillance System COVID-19 testing data, and the Index of Multiple Deprivation. COVID-19 vaccination records (National Immunisation Management System) are available within TPP primary care data. In March 2020, the Secretary of State for Health and Social Care used powers under the UK Health Service (Control of Patient Information) Regulations 2002 to require organizations to process confidential patient information for the purposes of protecting public health, providing health care services to the public, and monitoring and managing the COVID-19 outbreak and incidents of exposure; this sets aside the requirement for patient consent. Ethics approval for this study was obtained from the Health Research Authority and the University of Bristol’s Faculty of Health Sciences Ethics Committee. This study is reported in line with the Strengthening the Reporting of Observational Studies in Epidemiology (STROBE) reporting guideline.

We present main findings for depression and serious mental illness (composite of schizophrenia, schizoaffective disorder, bipolar disorder, and psychotic depression). We also examined general anxiety disorders, posttraumatic stress disorder, eating disorders, addiction, self-harm, and suicide, which are presented in eTables 5-7 and eFigure 4 in Supplement 1. Each outcome was defined using the earliest of a Systematized Nomenclature of Medicine–Clinical Terms code (a structured clinical vocabulary to record clinical events) in primary care; start of a hospital admission with an *International Statistical Classification of Diseases and Related Health Problems*, *Tenth Revision* (*ICD-10*) code in any position; or death with an *ICD-10 *code as the primary or underlying cause. We considered the first record only as individuals were removed from the cohort censored at this point. Codes indicating a relevant mental illness prior to the study period were captured by history covariates. Individuals with multiple mental illnesses were included in all relevant analyses with other mental illnesses captured by history covariates.

Date of COVID-19 was defined as the first of confirmed diagnosis recorded in primary care, positive SARS-COV-2 polymerase chain reaction or antigen test recorded in the Second Generation Surveillance System, start of a hospital admission with a confirmed diagnosis in any position, or death with SARS-COV-2 infection listed as the primary or underlying cause. People with a hospital admission record including a confirmed diagnosis in the primary position within 28 days of first COVID-19 were defined as having had hospitalization for COVID-19. All other COVID-19 diagnoses were defined as not hospitalized for COVID-19. The term *confirmed diagnosis* refers to diagnoses where the virus was identified by laboratory testing, irrespective of clinical symptom severity. We defined the date of confirmed COVID-19 diagnosis using the first record only, although additional secondary care information was used to classify COVID-19 as either leading to hospitalization or not. We did not consider multiple COVID-19 diagnoses within the study period as there is no agreed-on definition for COVID-19 reinfection using routinely collected data.

Covariates identified as potential confounders included age, sex, ethnicity, deprivation (using the Index of Multiple Deprivation—the official measure of relative deprivation for small areas in England defined by 7 domains, including income and barriers to housing and services), smoking status, care home residence, health care work, GP-patient interactions in 2019, and binary indicators for comorbidities (eTable 1 in Supplement 1).

### Study Population

Three cohorts were defined (eTable 2, eFigure 1 in Supplement 1). The pre–vaccine availability cohort was followed up from January 1, 2020 (baseline), until the earliest of December 14, 2021,^18^ date of outcome, date of deregistration, or date of death. Exposure was defined as recorded COVID-19 between baseline and the earliest date of eligibility for COVID-19 vaccination, date of first vaccination, and June 18, 2021 (when all adults became eligible for vaccination). Follow-up in the vaccinated cohort started at the later of June 1, 2021 (baseline), or 2 weeks after a second COVID-19 vaccination and ended at the earliest of December 14, 2021, date of outcome, date of deregistration, or date of death. The unvaccinated cohort had not received a COVID-19 vaccine by 12 weeks after they became eligible for vaccination. Follow-up started at the later of June 1, 2021 (baseline), or 12 weeks after vaccination eligibility and ended at the earliest of December 14, 2021, date of outcome, date of deregistration, or date of death. Eligibility criteria for each cohort are provided in the eMethods in Supplement 1. Individuals could potentially be followed up in all 3 cohorts but most often they were in the pre–vaccine availability cohort and either the vaccinated or unvaccinated cohort. The cohort definitions imply that diagnoses of mental illnesses after eligibility for vaccination in people who were not diagnosed with COVID-19 could be included in the comparison incidence rate calculations in both the pre–vaccine availability cohort and at most 1 of the vaccinated and unvaccinated cohorts. However, each COVID-19 diagnosis could be recorded in only 1 of the 3 cohorts, and therefore, each post–COVID-19 mental illness outcome could be included in only 1 of the 3 cohorts. Therefore, findings from each cohort are close to being statistically independent.

### Statistical Analyses

For each cohort, baseline characteristics were described, and numbers of outcome events, person-years of follow-up, and incidence rates (per 100 000 person-years) before and after all COVID-19 diagnoses, those leading to hospitalization, and those not leading to hospitalization were tabulated. Time to first event was analyzed for each outcome. Cox models were fitted with calendar time scale using the cohort-specific baseline as the origin. Hazard ratios (HRs) for follow-up after vs before or without COVID-19 were estimated, splitting follow-up into the day of COVID-19 diagnosis (day 0), the remainder of 1 to 4 weeks, and 5 to 28 weeks after COVID-19 for all cohorts and additionally 29 to 52 and 53 to 102 weeks after COVID-19 for the pre–vaccine availability cohort. For computational efficiency, we used sampling for analyses containing more than 4 000 000 people; we included all people with the outcome event, all people with the exposure (COVID-19 diagnosis), and a 10% random sample (for general anxiety, depression, and serious mental illness) or 20% random sample (for all other outcomes) of people who were not diagnosed with COVID-19 and in whom the outcome event was not recorded. We used inverse probability weights to adjust for the sampling and derived confidence intervals using robust standard errors. For each outcome and cohort, we estimated age- and sex-adjusted and maximally adjusted HRs including all covariates. Restricted cubic splines were used to account for age unless otherwise specified. All models were stratified by region to construct risk sets within region, accounting for between-region variation in the baseline hazard.

Subgroup analyses according to history of the outcome, age group, sex, ethnicity, and COVID-19 history were conducted for depression and serious mental illness. We calculated absolute excess risk 28 weeks after COVID-19, including outcomes recorded on the day of COVID-19 diagnosis (day 0) and weighted by the proportion of people in age and sex strata in the pre–vaccine availability cohort (eMethods in Supplement 1).

The study was conducted in Python version 3.8.10 (Python Software Foundation), R version 4.0.2 (R Foundation), and Stata/MP version 16.1 (StataCorp) according to a prespecified protocol. Our protocol, analysis code, and code lists are available.^19^ All outputs were subjected to OpenSAFELY disclosure controls, including rounding where appropriate.^20^ Data were analyzed from July 2022 to June 2024.

## Results

The pre–vaccine availability cohort included 18 648 606 people (9 363 710 [50.2%] female and 9 284 896 [49.8%] male; median [IQR] age, 49 [34-64] years) 1 012 335 of whom had COVID-19 (Table 1; eFigure 2 in the Supplement). The cohort included 1 191 793 (6.4%) Black individuals; 217 132 (1.2%) South Asian individuals; 14 865 866 (79.7%) White individuals; 423 111 individuals (2.3%) of mixed ethnicity; and 400 437 of other ethnicities (2.1%), consolidated for disclosure control; and 1 550 267 individuals (8.3%) for whom ethnicity data were missing. The vaccinated cohort included 14 035 286 individuals (median [IQR] age, 53 [38-67] years; 7 308 556 [52.1%] female and 6 726 730 [47.9%] male; 789 476 [5.6%] Black, 128 514 [0.9%] South Asian, 11 752 297 [83.7%] White, 237 383 [1.7%] mixed, 789 476 [5.6%] other, and 910 299 [6.5%] missing), 866 469 of whom had COVID-19. The unvaccinated cohort included 3 242 215 people (median [IQR] age, 35 [27-46] years; 1 363 401 [42.1%] female and 1 878 814 [57.9%] male; 325 199 [10%] Black, 81 017 [2.5%] South Asian, 2 025 492 [62.5%] White, 190 874 [5.9%] mixed, 173 014 [5.3%] other, 446 619 [13.8%] missing), 149 745 of whom had COVID-19. Differences in demographic characteristics between these cohorts reflect factors associated with COVID-19 vaccine uptake.^21^ eTable 3 in Supplement 1 summarizes participants’ medical history by cohort. Rates of COVID-19 differed by cohort (Table 1). Higher rates were observed in cohorts followed up when the Delta variant was dominant (vaccinated: 915 per 100 000 person years and unvaccinated: 1274 per 100 000 person years) than when the wild-type or Alpha variants were dominant (pre–vaccine availability: 308 per 100 000 person years). Of the 18 648 606 individuals in the pre–vaccine availability cohort, 12 969 492 (69.5%) were subsequently followed up in the vaccinated cohort and 2 843 514 (15.2%) were subsequently followed up in the unvaccinated cohort (eFigure 3 in Supplement 1). Of 17 121 348 individuals included in either the unvaccinated or vaccinated cohort, 156 144 (0.91%) were included in both.

**Table 1.** Patient Characteristics by Cohort

Characteristic	Pre–vaccine availability		Vaccinated		Unvaccinated	
	No. (%)	COVID-19 diagnoses	No. (%)	COVID-19 diagnoses	No. (%)	COVID-19 diagnoses
Total, No.	18 648 606	1 012 335	14 035 286	866 469	3 242 215	149 745
Sex						
Female	9 363 710 (50.2)	543 513	7 308 556 (52.1)	481 581	1 363 401 (42.1)	79 179
Male	9 284 896 (49.8)	468 825	6 726 730 (47.9)	384 891	1 878 814 (57.9)	70 569
Age range, y						
18-29	3 278 187 (17.6)	244 839	1 760 740 (12.5)	94 533	981 925 (30.3)	40 131
30-39	3 225 998 (17.3)	203 931	1 958 328 (14)	149 193	966 470 (29.8)	50 655
40-49	3 059 107 (16.4)	190 065	2 206 560 (15.7)	236 229	612 180 (18.9)	33 429
50-59	3 264 621 (17.5)	184 371	2 716 818 (19.4)	204 009	376 272 (11.6)	16 827
60-69	2 552 894 (13.7)	92 931	2 316 757 (16.5)	105 645	186 402 (5.7)	5625
70-79	2 066 329 (11.1)	51 573	1 980 447 (14.1)	54 057	80 546 (2.5)	1947
80-89	989 819 (5.3)	31 893	909 927 (6.5)	18 327	30 138 (0.9)	903
≥90	211 651 (1.1)	12 717	185 709 (1.3)	4479	8282 (0.3)	225
Ethnicity^a^						
Black	1 191 793 (6.4)	118 365	789 476 (5.6)	41 457	325 199 (10)	9609
South Asian	217 132 (1.2)	14 241	128 514 (0.9)	7521	81 017 (2.5)	3867
White	14 865 866 (79.7)	773 745	11 752 297 (83.7)	748 743	2 025 492 (62.5)	115 449
Mixed	423 111 (2.3)	20 301	237 383 (1.7)	10 641	190 874 (5.9)	4035
Other^b^	400 437 (2.1)	27 069	217 317 (1.5)	9585	173 014 (5.3)	7641
Missing	1 550 267 (8.3)	58 611	910 299 (6.5)	48 513	446 619 (13.8)	9141
IMD quintile						
1	3 574 653 (19.2)	247 365	2 273 863 (16.2)	135 945	958 877 (29.6)	45 795
2	3 690 808 (19.8)	219 063	2 617 493 (18.6)	158 889	775 318 (23.9)	35 445
3	4 042 896 (21.7)	203 055	3 111 723 (22.2)	186 855	652 380 (20.1)	29 619
4	3 809 548 (20.4)	183 015	3 065 889 (21.8)	191 499	498 458 (15.4)	22 515
5	3 530 701 (18.9)	159 837	2 966 318 (21.1)	193 275	357 182 (11)	16 371
Smoking						
Never	759 965 (4.1)	40 677	428 549 (3.1)	16 419	370 770 (11.4)	8415
Formerly	8 568 556 (45.9)	504 843	6 540 674 (46.6)	422 337	1 348 307 (41.6)	59 631
Currently	6 137 311 (32.9)	329 373	5 106 300 (36.4)	333 675	645 817 (19.9)	43 887
Missing	3 182 774 (17.1)	137 439	1 959 763 (14)	94 035	877 321 (27.1)	37 815
Region						
East	4 305 743 (23.1)	227 421	3 281 525 (23.4)	182 847	731 831 (22.6)	33 825
East Midlands	3 263 511 (17.5)	191 925	2 474 916 (17.6)	161 259	527 562 (16.3)	28 815
London	1 245 392 (6.7)	67 791	728 567 (5.2)	37 287	459 504 (14.2)	10 977
North East	889 507 (4.8)	60 369	663 394 (4.7)	48 891	137 238 (4.2)	7239
North West	1 658 370 (8.9)	109 887	1 270 319 (9.1)	88 227	220 722 (6.8)	12 045
South East	1 249 245 (6.7)	50 373	969 482 (6.9)	57 105	195 663 (6)	8853
South West	2 655 179 (14.2)	80 649	2 207 972 (15.7)	133 143	331 576 (10.2)	17 871
West Midlands	760 648 (4.1)	56 397	499 564 (3.6)	29 931	181 504 (5.6)	8223
Yorkshire	2 621 011 (14.1)	167 505	1 939 547 (13.8)	127 767	456 615 (14.1)	21 891
Care home resident	90 076 (0.5)	15 309	57 532 (0.4)	2835	3014 (0.1)	105

Abbreviation: IMD, Index of Multiple Deprivation (lower indicates more deprived).

^a^ Ethnicity data were reported because disparities in COVID-19 outcomes by ethnic group have been reported.^17^

^b^ Other ethnicity groups were consolidated for disclosure control.

The incidence of mental illnesses was higher after COVID-19 than before or without COVID-19 (Table 2). Between-cohort differences in the incidence of mental illnesses in the absence of COVID-19 likely reflect both demographic differences and changes in diagnostic practices and access to health care during the pandemic. The highest incidence rates were after hospitalization for COVID-19. Depression was the most common outcome with 1 329 270, 352 944, and 57 810 diagnoses in the pre–vaccine availability, vaccinated, and unvaccinated cohorts, respectively. The corresponding diagnoses of serious mental illness were 397 368, 88 500, and 18 726. Separating individuals by their history of the outcome, incidence of mental illnesses after COVID-19 was greater in those with than without history (eTable 4 in Supplement 1).

**Table 2.** Mental Illness Events Following Diagnosis of COVID-19 by Cohort, Overall, and by COVID-19 Severity

Outcome	Pre–vaccine availability (n = 18 648 606)		Vaccinated (n = 14 035 286)		Unvaccinated (n = 3 242 215)	
	Event/person-years	Incidence rate	Event/person-years	Incidence rate	Event/person-years	Incidence rate
Depression						
No COVID-19	1 278 435/33 058 669	3867	341 811/6 293 635	5431	54 903/1 207 059	4548
Hospitalized for COVID-19	6333/41 555	15 240	1395/2426	57 491	849/1636	51 902
Not hospitalized for COVID-19	44 499/897 194	4960	9741/156 195	6236	2061/25 034	8233
Serious mental illness						
No COVID-19	382 305/33 963 171	1126	85 917/6 359 387	1351	17 985/1 215 060	1480
Hospitalized for COVID-19	1653/47 219	3501	213/2705	7874	165/1796	9185
Not hospitalized for COVID-19	13 407/945 179	1418	2367/158 999	1489	573/25 466	2250
General anxiety disorders						
No COVID-19	937 383/33 434 332	2804	228 873/6 323 675	3619	40 491/1 210 498	3345
Hospitalized for COVID-19	4887/43 706	11 182	1047/2531	41 374	891/1639	54 368
Not hospitalized for COVID-19	35 751/913 859	3912	7023/157 377	4463	1719/25 148	6835
Posttraumatic stress disorder						
No COVID-19	37 083/34 312 260	108	8307/6 378 443	130	2823/1 218 521	232
Hospitalized for COVID-19	309/49 010	630	63/2750	2291	63/1824	3453
Not hospitalized for COVID-19	1335/963 447	139	237/159 781	148	93/25 602	363
Eating disorders						
No COVID-19	19 347/34 329 162	56	4689/6 379 405	74	939/1 218 942	77
Hospitalized for COVID-19	63/49 272	128	9/2763	326	15/1834	818
Not hospitalized for COVID-19	807/964 120	84	135/159 806	84	33/25 623	129
Addiction						
No COVID-19	38 289/34 308 296	112	5589/6 379 097	88	4233/1 218 061	348
Hospitalized for COVID-19	171/49 136	348	21/2760	761	51/1825	2794
Not hospitalized for COVID-19	921/963 714	96	105/159 822	66	87/25 603	340
Self-harm						
No COVID-19	90 453/34 259 659	264	15 543/6 376 874	244	5097/1 218 061	418
Hospitalized for COVID-19	255/48 990	521	27/2759	979	15/1835	817
Not hospitalized for COVID-19	3087/960 343	321	525/159 685	329	147/25 588	574
Suicide						
No COVID-19	3651/34 347 490	11	537/6 380 535	8	189/1 219 140	16
Hospitalized for COVID-19	9/49 355	18	3/2765	108	0/1839	0
Not hospitalized for COVID-19	87/965 254	9	15/15 9864	9	9/25 633	35

### Comparisons of Event Rates After Diagnosis of COVID-19 vs Before or Without COVID-19

Maximally adjusted HRs (aHRs) comparing the incidence of each outcome after COVID-19 with the incidence before or without COVID-19 did not differ substantially from the age- and sex-adjusted HRs (eFigure 4 in Supplement 1). The incidence of all outcomes was extremely high on day 0 (eTable 5 in Supplement 1). The incidence of most outcomes was elevated during the remainder of 1 to 4 weeks after COVID-19, compared with before or without COVID-19, in each cohort. For all figures, aHRs are plotted against the median time from date of COVID-19 diagnosis to date of outcome in each cohort.

### Depression

**Figure 1.**
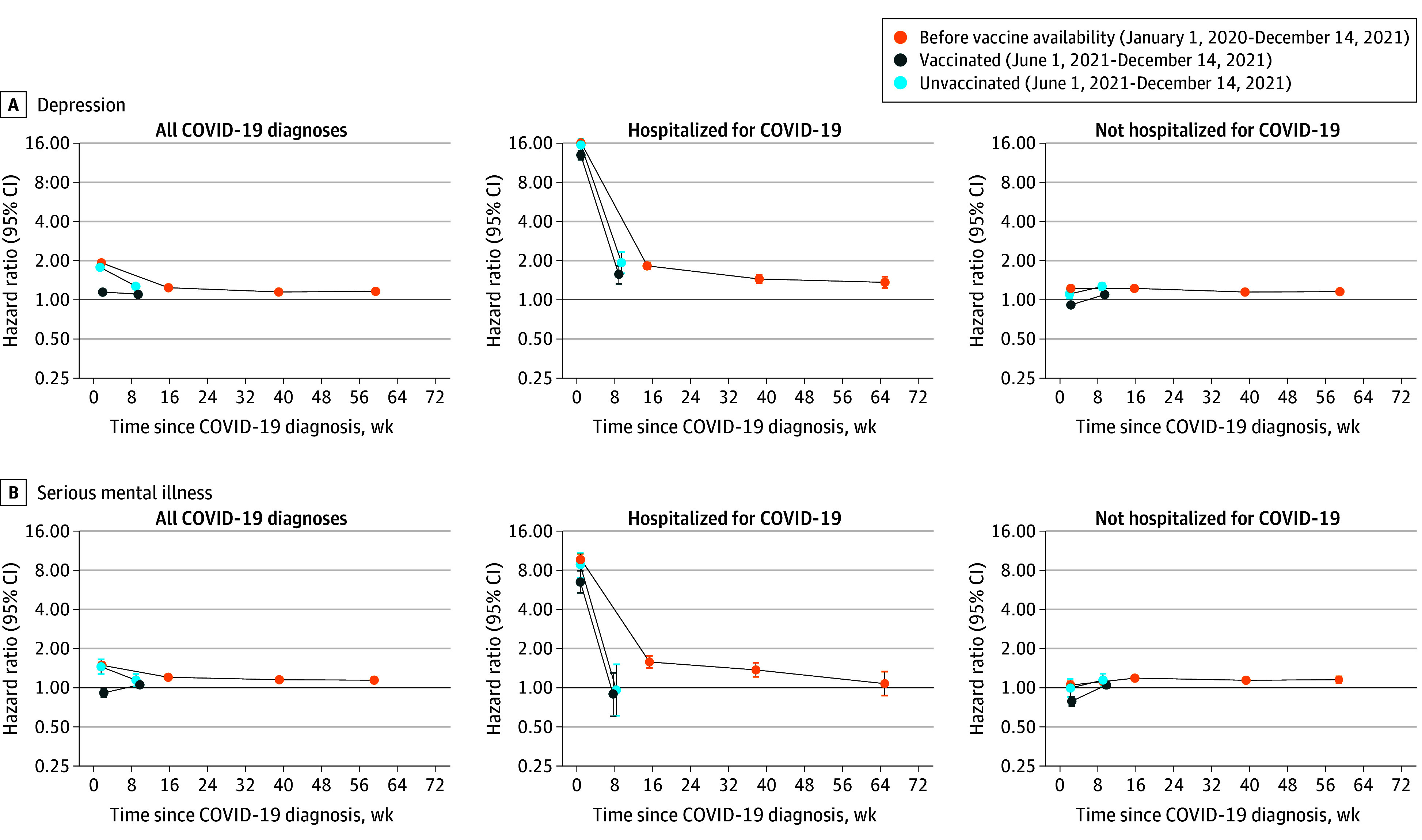
Maximally Adjusted Hazard Ratios and 95% CIs for Depression and Serious Mental Illness Following Diagnosis of COVID-19, Overall, and by COVID-19 Severity

Incidence of depression was elevated during weeks 1 through 4 after COVID-19, compared with before or without COVID-19, in the pre–vaccine availability and unvaccinated cohorts (aHR, 1.93; 95% CI, 1.88-1.98 and aHR, 1.79; 95% CI, 1.68-1.90, respectively) and, to a lesser extent, the vaccinated cohort (aHR, 1.16; 95% CI, 1.12-1.20) (Figure 1; eTable 5 in Supplement 1). Incidence remained elevated during weeks 5 through 28 in the vaccinated and unvaccinated cohorts (aHR, 1.11; 95% CI, 1.08-1.14 and aHR, 1.28; 95% CI, 1.21-1.36, respectively) and up to weeks 53 through 102 in the pre–vaccine availability cohort (aHR, 1.17; 95% CI, 1.14-1.21). aHRs during weeks 1 through 4 were considerably higher after COVID-19 with hospitalization (pre–vaccine availability: 16.3; 95% CI, 15.6-17.0; vaccinated: 12.9; 95% CI, 12.0-14.0; unvaccinated: 15.6; 95% CI, 13.9-17.4) than without hospitalization (pre–vaccine availability: 1.22; 95% CI, 1.18-1.27; vaccinated: 0.92; 95% CI, 0.88-0.95; unvaccinated: 1.11; 95% CI, 1.02-1.20) (Figure 1; eTables 6 and 7 in Supplement 1). In the pre–vaccine availability cohort, aHRs remained higher after COVID-19 with hospitalization than without hospitalization throughout follow-up.

### Serious Mental Illness

Incidence of serious mental illness was elevated during weeks 1 through 4 after COVID-19, compared with before or without COVID-19, in the pre–vaccinated and unvaccinated cohorts (aHR, 1.49; 95% CI, 1.41-1.57 and aHR, 1.45; 95% CI, 1.27-1.65, respectively) (Figure 1; eTable 5 in Supplement 1). However, incidence was lower during weeks 1 through 4 in the vaccinated cohort (aHR, 0.91; 95% CI, 0.85-0.98). Incidence remained slightly elevated during weeks 5 through 28 in the vaccinated and unvaccinated cohorts (aHR, 1.05; 95% CI, 1.00-1.11 and aHR, 1.14; 95% CI, 1.02-1.27) and up to weeks 53 through 102 in the pre–vaccine availability cohort (aHR, 1.14; 95% CI, 1.08-1.21). Incidence of serious mental illness during weeks 1 through 4 was considerably higher after COVID-19 with hospitalization (pre–vaccine availability: aHR, 9.71; 95% CI, 8.80-10.7; vaccinated: aHR, 6.52; 95% CI, 5.36-7.93; unvaccinated: aHR, 8.75; 95% CI, 7.01-10.9) than after COVID-19 without hospitalization (pre–vaccine availability: aHR, 1.05; 95% CI, 0.98-1.12; vaccinated: aHR, 0.79; 95% CI, 0.73-0.86; unvaccinated: aHR, 1.00; 95% CI, 0.85-1.17) (Figure 1; eTables 6 and 7 in Supplement 1).

### Subgroup Analyses

**Figure 2.**
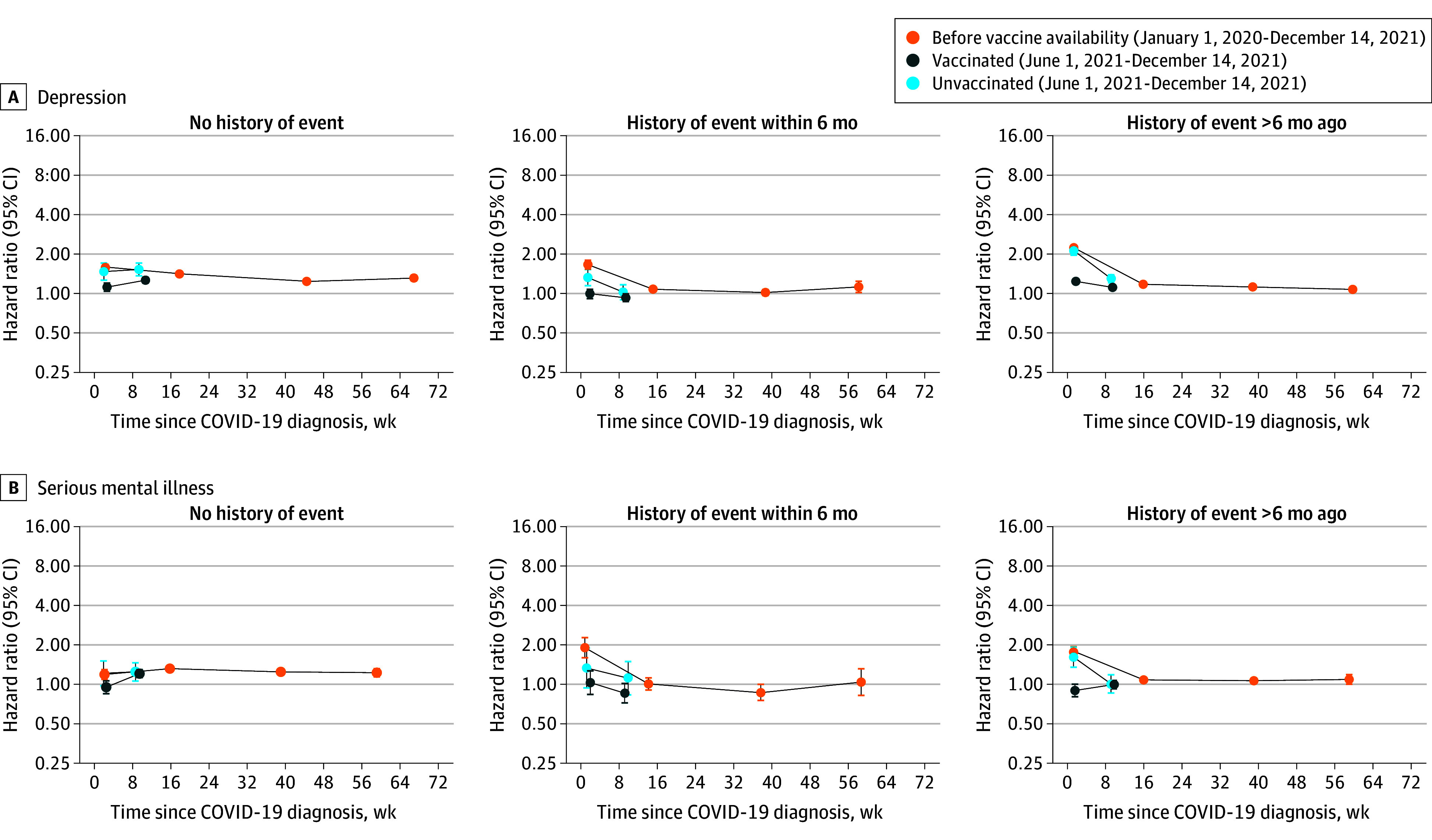
Maximally Adjusted Hazard Ratios and 95% CIs for Depression and Serious Mental Illness Following Diagnosis of COVID-19 by History of the Outcome

Incidence of depression was highest during weeks 1 through 4 after COVID-19, vs before or without COVID-19, for people with history of the outcome more than 6 months ago (Figure 2; eTables 8-10 in Supplement 1). However, incidence of serious mental illness was highest during weeks 1 through 4 after COVID-19 for people with history of the outcome within 6 months for the pre–vaccine availability (aHR, 1.90; 95% CI, 1.60-2.27) and vaccinated cohorts (aHR, 1.03; 95% CI, 0.84-1.27). Incidences of depression and serious mental illness after COVID-19, vs before or without COVID-19, were similar in people with and without a history of COVID-19 (eFigure 5 and eTable 11 in Supplement 1). Incidence of depression during weeks 1 through 4 and 5 through 28 and for serious mental illness across all time periods were greater in the age groups 60 years and older than in the age groups 60 years and younger (eFigure 6 and eTables 12-15 in Supplement 1). Incidences of depression and serious mental illness were marginally higher for men than women during weeks 1 through 4 after COVID-19 (eFigure 7 and eTables 16 and 17 in Supplement 1). Incidences of depression and serious mental illness after COVID-19 were generally comparable between ethnic groups, when they could be estimated (eFigure 8, eTables 18-22 in Supplement 1).

### Other Mental Illnesses

**Figure 3.**
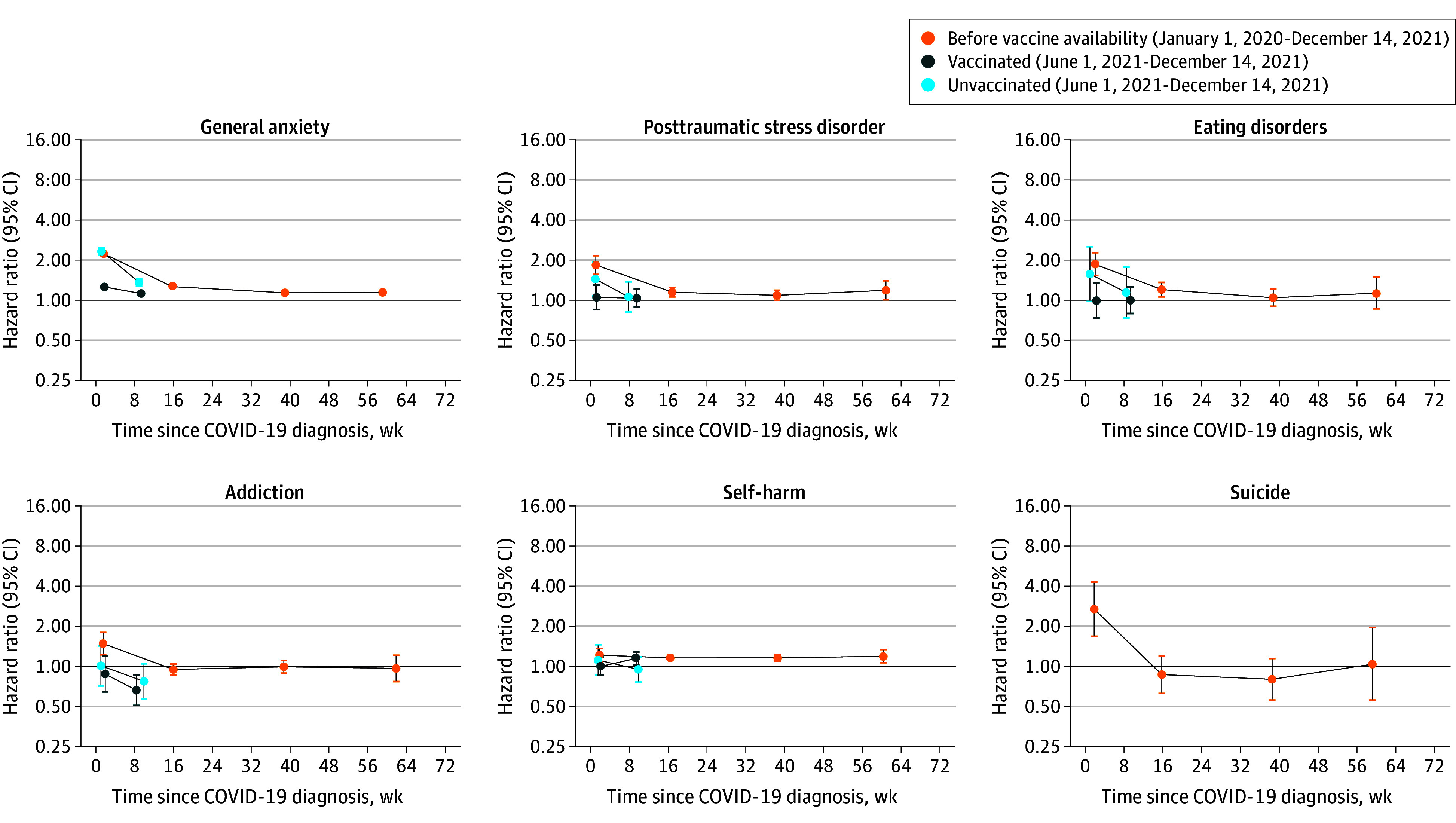
Maximally Adjusted Hazard Ratios and 95% Confidence Intervals for Other Mental Illness Events Following Diagnosis of COVID-19

Incidences of other mental illnesses were broadly similar to those of depression and serious mental illness, both overall (Figure 3, eTable 5 in Supplement 1) and for COVID-19 with and without hospitalization (eTables 6 and 7 in Supplement 1). An exception was that incidence of posttraumatic stress disorder after COVID-19 with hospitalization, vs before or without COVID-19, was higher during weeks 1 through 4 in the vaccinated cohort than the other cohorts (pre–vaccine availability: aHR, 20.1; 95% CI, 15.8-25.6; vaccinated: aHR, 27.3; 95% CI, 20.3-36.6; unvaccinated: aHR, 13.3; 95% CI, 8.00-22.2). This pattern was not present for COVID-19 without hospitalization or overall.

### Absolute Excess Risk

Estimated excess risks of depression 28 weeks after COVID-19, standardized to the age and sex distribution of the pre–vaccine availability cohort, were 1033, 451, and 1008 per 100 000 people in the pre–vaccine availability, vaccinated, and unvaccinated cohorts, respectively (eFigure 9 and eTable 23 in Supplement 1). The equivalent estimated excess risks of serious mental illness were 235, 53, and 209 per 100 000 people. Many of the estimated excess events occurred on the day of COVID-19 diagnosis (day 0).

## Discussion

In this cohort study of more than 18 million people with up to 2 years of follow-up, rates of most mental illnesses were markedly elevated during weeks 1 through 4 after COVID-19 compared with before or without COVID-19. This elevation was less marked in people who were vaccinated before COVID-19. In people with COVID-19 before vaccination was available, incidence of mental illnesses remained elevated more than 28 weeks after diagnosis, particularly in people who were hospitalized. In subgroup analyses according to history of the outcome, associations 1 through 4 weeks after COVID-19 were greater in those with than without history. Subgroup analyses also suggested stronger associations in older age groups and in men. The association of COVID-19 with mental illnesses did not differ markedly between ethnic groups.

Attenuation of adverse effects of COVID-19 on mental illnesses in the vaccinated may be explained by reduced disease severity due to vaccination.^22^ Potential mechanisms include reduced systemic inflammation and psychological benefits of vaccination, such as reduced concern about COVID-19 and increased social engagement.^23^ A previous study^3^ found that associations varied by COVID-19 severity, with poorer mental illness outcomes only found among those who were bedridden with COVID-19.

Rates of mental illness outcomes declined with increasing time since COVID-19, although incidence remained elevated up to a year after COVID-19 with hospitalization in the pre–vaccine availability era. Previous findings have been mixed, with a review reporting no clear long-term associations between COVID-19 and mental illness,^24^ while a multicohort study found little evidence of attenuation over time.^1^ Persisting associations of COVID-19 with mental illnesses could partly reflect ongoing impacts of post–COVID-19 condition.^25,26^

Consistent with previous research,^1^ we found stronger associations between COVID-19 and mental illnesses among older age groups. This is likely to reflect their increased risk of severe COVID-19 and resulting increased anxiety about its outcomes. The association between COVID-19 and mental illnesses was slightly stronger among men, who have been found to be at greater risk of severe mental illness outcomes than women.^27^ These patterns contrast with the wider impacts of the pandemic on mental health, which have been found to be greatest in adults aged 25 to 44 years, women, and those with higher educational degrees.^28^ This indicates that mechanisms linking COVID-19 and mental health may differ from those underpinning wider pandemic effects.

Our findings highlight the wider public health benefits of vaccination. Prior mental illness may influence vaccine uptake, highlighting the importance of actively encouraging vaccination of people with mental health difficulties.^21,29,30^ Our analyses suggested that the adverse associations of COVID-19 with mental illnesses were greater prior to the availability of vaccination. This may reflect greater uncertainty and public concern around outcomes of COVID-19 and treatment effectiveness at the beginning of the pandemic.

### Strengths and Limitations

Strengths of this study include the large sample size, the detailed linked electronic health record data, the relatively long duration of follow-up, and the opportunity to account for vaccination. We also note several limitations. First, electronic health records are routinely collected data for health care provision and so only capture conditions diagnosed and recorded by the health care professional rather than true incidence in the population. Unvaccinated people may have been less likely to contact health services and to test for SARS-CoV-2 infection, leading to underestimated effects. People with recorded COVID-19, particularly COVID-19 with hospitalization, may be more likely to have mental illnesses recorded due to greater contact with health services. This may underpin the particularly high HRs observed initially, especially in those hospitalized, and the rapid fall as service contact is likely highest early after diagnosis. However, this is unlikely to fully explain adverse effects, given the persistent elevation of incidence of mental illnesses following COVID-19 with hospitalization and the variation across mental illnesses. Also, people with prior recorded mental health diagnoses may not have them coded at every visit, even if their mental health had deteriorated due to COVID-19. Additionally, data on mental health are generally incomplete, as they do not include mental health services data or National Health Service Talking Therapies (formerly Improving Access to Psychological Therapies), to which patients can self-refer. This is relevant to the present study as those with more serious COVID-19 are more likely to be in contact with health services and therefore may be more likely to report symptoms, while those not in contact may not seek help or may use other routes that are not captured. Again, this relates to the particularly high HRs observed initially, which may reflect the recording of both COVID-19 and mental illnesses during the same consultation. Furthermore, we could only assess COVID-19 severity according to hospitalization and did not consider the potential role of repeated infections. We cannot exclude the possibility of unmeasured confounding, although we controlled for a wide range of demographic characteristics and prior morbidities. More sophisticated methods for potential confounding exist and may have been more appropriate here, although these more computationally intensive methods were not feasible given the size of dataset analyzed.^31^ A previous study^2^ found that the mental health impacts of COVID-19 were less apparent when using a negative control group (a group of individuals whose SARS-CoV-2 polymerase chain reaction or antigen test result had a negative result), suggesting that observed associations may have been, at least in part, due to unmeasured confounding related to testing behavior. We were not able to include a negative control group to explore this as the available test data did not include negative results. Additionally, other viruses may have implications for mental illnesses. Our findings may therefore reflect a phenomenon that occurs after many viruses rather than specifically SARS-CoV-2.

## Conclusions

The findings in this study add to a growing body of evidence highlighting the increased risk of mental illnesses following COVID-19 diagnosis, with stronger associations found in relation to nonvaccination and more severe COVID-19 disease and longer-term associations relating mainly to new-onset mental illnesses. This has important implications for public health and mental health service provision, as serious mental illnesses are associated with more intensive health care needs and longer-term health and other adverse effects. Our results highlight the importance COVID-19 vaccination in the general population and particularly among those with mental illnesses, who may be at higher risk of both SARS-CoV-2 infection and adverse outcomes following COVID-19.

## Notes

Supplement 1. eMethods eTable 1. Summary of covariate definitions eTable 2. Summary of cohort characteristics eTable 3. Medical history of people in the pre-vaccine availability, vaccinated and unvaccinated cohorts eTable 4. Mental illness events following diagnosis of COVID-19 in the pre-vaccine availability, vaccinated and unvaccinated cohorts, by prior history of the outcome eTable 5. Maximally adjusted hazard ratios and 95% CIs for mental illness events following diagnosis of COVID-19 in the pre-vaccine availability, vaccinated and unvaccinated cohorts eTable 6. Maximally adjusted hazard ratios and 95% CIs for mental illness events following hospitalised COVID-19 in the pre-vaccine availability, vaccinated and unvaccinated cohorts eTable 7. Maximally adjusted hazard ratios and 95% Cis for mental illness events following non-hospitalised COVID-19 in the pre-vaccine availability, vaccinated and unvaccinated cohorts eTable 8. Maximally adjusted hazard ratios and 95% CIs for mental illness events following diagnosis of COVID-19 in the pre-vaccine availability, vaccinated and unvaccinated cohorts, in people with no prior history of the outcome eTable 9. Maximally adjusted hazard ratios and 95% CIs for mental illness events following diagnosis of COVID-19 in the pre-vaccine availability, vaccinated and unvaccinated cohorts, in people with prior history of the outcome, more than six months ago eTable 10. Maximally adjusted hazard ratios and 95% CIs for mental illness events following diagnosis of COVID-19 in the pre-vaccine availability, vaccinated and unvaccinated cohorts, in people with prior history of the outcome, within six months eTable 11. Maximally adjusted hazard ratios and 95% CIs for mental illness events following diagnosis of COVID-19 in the pre-vaccine availability, vaccinated and unvaccinated cohorts, in people with history of COVID-19 eTable 12. Maximally adjusted hazard ratios and 95% CIs for mental illness events following diagnosis of COVID-19 in the pre-vaccine availability, vaccinated and unvaccinated cohorts for age group 18-39 eTable 13. Maximally adjusted hazard ratios and 95% CIs for mental illness events following diagnosis of COVID-19 in the pre-vaccine availability, vaccinated and unvaccinated cohorts for age group 40-59 eTable 14. Maximally adjusted hazard ratios and 95% CIs for mental illness events following diagnosis of COVID-19 in the pre-vaccine availability, vaccinated and unvaccinated cohorts for age group 60-79 eTable 15. Maximally adjusted hazard ratios and 95% CIs for mental illness events following diagnosis of COVID-19 in the pre-vaccine availability, vaccinated and unvaccinated cohorts for age group 80-110 eTable 16. Maximally adjusted hazard ratios and 95% CIs for mental illness events following diagnosis of COVID-19 in the pre-vaccine availability, vaccinated and unvaccinated cohorts for women eTable 17. Maximally adjusted hazard ratios and 95% CIs for mental illness events following diagnosis of COVID-19 in the pre-vaccine availability, vaccinated and unvaccinated cohorts for men eTable 18. Maximally adjusted hazard ratios and 95% CIs for mental illness events following diagnosis of COVID-19 in the pre-vaccine availability, vaccinated and unvaccinated cohorts for White ethnicity eTable 19. Maximally adjusted hazard ratios and 95% CIs for mental illness events following diagnosis of COVID-19 in the pre-vaccine availability, vaccinated and unvaccinated cohorts for South Asian ethnicity eTable 20. Maximally adjusted hazard ratios and 95% CIs for mental illness events following diagnosis of COVID-19 in the pre-vaccine availability, vaccinated and unvaccinated cohorts for Black ethnicity eTable 21. Maximally adjusted hazard ratios and 95% CIs for mental illness events following diagnosis of COVID-19 in the pre-vaccine availability, vaccinated and unvaccinated cohorts for Other ethnicity eTable 22. Maximally adjusted hazard ratios and 95% CIs for mental illness events following diagnosis of COVID-19 in the pre-vaccine availability, vaccinated and unvaccinated cohorts for Mixed ethnicity eTable 23. Excess events per 100,000 people at 28 weeks post-COVID-19 in the pre-vaccine availability, vaccinated and unvaccinated cohorts eFigure 1. COVID-19 cases over time eFigure 2. Diagram showing cohort construction eFigure 3. Venn Diagram showing cohort overlap eFigure 4. Maximally and minimally adjusted hazard ratios and 95% CIs for mental illness events following diagnosis of COVID-19 in the pre-vaccine availability, vaccinated and unvaccinated cohorts. Events on the day of COVID-19 diagnosis (day 0) were excluded eFigure 5. Maximally adjusted hazard ratios and 95% CIs for depression and serious mental illness following diagnosis of COVID-19 in the vaccinated and unvaccinated cohorts, by history of COVID-19. Events on the day of COVID-19 diagnosis (day 0) were excluded eFigure 6. Maximally adjusted hazard ratios and 95% CIs for depression and serious mental illness following diagnosis of COVID-19 in the pre-vaccine availability, vaccinated and unvaccinated cohorts, by age group. Events on the day of COVID-19 diagnosis (day 0) were excluded eFigure 7. Maximally adjusted hazard ratios and 95% CIs for depression and serious mental illness following diagnosis of COVID-19 in the pre-vaccine availability, vaccinated and unvaccinated cohorts, by sex. Events on the day of COVID-19 diagnosis (day 0) were excluded eFigure 8. Maximally adjusted hazard ratios and 95% CIs for depression and serious mental illness following diagnosis of COVID-19 in the pre-vaccine availability, vaccinated and unvaccinated cohorts, by ethnicity. Events on the day of COVID-19 diagnosis (day 0) were excluded eFigure 9. Absolute excess risk up to 28 weeks for depression and serious mental illness following diagnosis of COVID-19 in the pre-vaccine availability, vaccinated and unvaccinated cohorts

Supplement 2. Members of the Longitudinal Health and Wellbeing COVID-19 National Core Study

Supplement 3. Data sharing statement
