# Supplementary material for: COVID-19 and Mental Illnesses in Vaccinated and Unvaccinated People
Source: JAMA Psychiatry. 2024 Aug 21;81(11):1071–80. doi: 10.1001/jamapsychiatry.2024.2339 (PMC11339697; doi:10.1001/jamapsychiatry.2024.2339)

Supplemental Online Content

Walker VM, Patalay P, Cuitun Coronado JI, et al; the Longitudinal Health and Wellbeing COVID-19 National Core Study group. COVID-19 and mental illness in vaccinated and unvaccinated people. *JAMA Psychiatry*. Published online August 21, 2024. doi:10.1001/jamapsychiatry.2024.2339

eMethods
eTable 1. Summary of covariate definitions
eTable 2. Summary of cohort characteristics
eTable 3. Medical history of people in the pre-vaccine availability, vaccinated and unvaccinated cohorts
eTable 4. Mental illness events following diagnosis of COVID-19 in the pre-vaccine availability, vaccinated and unvaccinated cohorts, by prior history of the outcome
eTable 5. Maximally adjusted hazard ratios and 95% CIs for mental illness events following diagnosis of COVID-19 in the pre-vaccine availability, vaccinated and unvaccinated cohorts
eTable 6. Maximally adjusted hazard ratios and 95% CIs for mental illness events following hospitalised COVID-19 in the pre-vaccine availability, vaccinated and unvaccinated cohorts
eTable 7. Maximally adjusted hazard ratios and 95% Cis for mental illness events following non-hospitalised COVID-19 in the pre-vaccine availability, vaccinated and unvaccinated cohorts
eTable 8. Maximally adjusted hazard ratios and 95% CIs for mental illness events following diagnosis of COVID-19 in the pre-vaccine availability, vaccinated and unvaccinated cohorts, in people with no prior history of the outcome
eTable 9. Maximally adjusted hazard ratios and 95% CIs for mental illness events following diagnosis of COVID-19 in the pre-vaccine availability, vaccinated and unvaccinated cohorts, in people with prior history of the outcome, more than six months ago
eTable 10. Maximally adjusted hazard ratios and 95% CIs for mental illness events following diagnosis of COVID-19 in the pre-vaccine availability, vaccinated and unvaccinated cohorts, in people with prior history of the outcome, within six months
eTable 11. Maximally adjusted hazard ratios and 95% CIs for mental illness events following diagnosis of COVID-19 in the pre-vaccine availability, vaccinated and unvaccinated cohorts, in people with history of COVID-19
eTable 12. Maximally adjusted hazard ratios and 95% CIs for mental illness events following diagnosis of COVID-19 in the pre-vaccine availability, vaccinated and unvaccinated cohorts for age group 18-39
eTable 13. Maximally adjusted hazard ratios and 95% CIs for mental illness events following diagnosis of COVID-19 in the pre-vaccine availability, vaccinated and unvaccinated cohorts for age group 40-59
eTable 14. Maximally adjusted hazard ratios and 95% CIs for mental illness events following diagnosis of COVID-19 in the pre-vaccine availability, vaccinated and unvaccinated cohorts for age group 60-79
eTable 15. Maximally adjusted hazard ratios and 95% CIs for mental illness events following diagnosis of COVID-19 in the pre-vaccine availability, vaccinated and unvaccinated cohorts for age group 80-110
eTable 16. Maximally adjusted hazard ratios and 95% CIs for mental illness events following diagnosis of COVID-19 in the pre-vaccine availability, vaccinated and unvaccinated cohorts for women
eTable 17. Maximally adjusted hazard ratios and 95% CIs for mental illness events following diagnosis of COVID-19 in the pre-vaccine availability, vaccinated and unvaccinated cohorts for men

© 2024 Walker VM et al. *JAMA Psychiatry.*

eTable 18. Maximally adjusted hazard ratios and 95% CIs for mental illness events following diagnosis of COVID-19 in the pre-vaccine availability, vaccinated and unvaccinated cohorts for White ethnicity
eTable 19. Maximally adjusted hazard ratios and 95% CIs for mental illness events following diagnosis of COVID-19 in the pre-vaccine availability, vaccinated and unvaccinated cohorts for South Asian ethnicity
eTable 20. Maximally adjusted hazard ratios and 95% CIs for mental illness events following diagnosis of COVID-19 in the pre-vaccine availability, vaccinated and unvaccinated cohorts for Black ethnicity
eTable 21. Maximally adjusted hazard ratios and 95% CIs for mental illness events following diagnosis of COVID-19 in the pre-vaccine availability, vaccinated and unvaccinated cohorts for Other ethnicity
eTable 22. Maximally adjusted hazard ratios and 95% CIs for mental illness events following diagnosis of COVID-19 in the pre-vaccine availability, vaccinated and unvaccinated cohorts for Mixed ethnicity
eTable 23. Excess events per 100,000 people at 28 weeks post-COVID-19 in the pre-vaccine availability, vaccinated and unvaccinated cohorts
eFigure 1. COVID-19 cases over time
eFigure 2. Diagram showing cohort construction
eFigure 3. Venn Diagram showing cohort overlap
eFigure 4. Maximally and minimally adjusted hazard ratios and 95% CIs for mental illness events following diagnosis of COVID-19 in the pre-vaccine availability, vaccinated and unvaccinated cohorts. Events on the day of COVID-19 diagnosis (day 0) were excluded
eFigure 5. Maximally adjusted hazard ratios and 95% CIs for depression and serious mental illness following diagnosis of COVID-19 in the vaccinated and unvaccinated cohorts, by history of COVID-19. Events on the day of COVID-19 diagnosis (day 0) were excluded
eFigure 6. Maximally adjusted hazard ratios and 95% CIs for depression and serious mental illness following diagnosis of COVID-19 in the pre-vaccine availability, vaccinated and unvaccinated cohorts, by age group. Events on the day of COVID-19 diagnosis (day 0) were excluded
eFigure 7. Maximally adjusted hazard ratios and 95% CIs for depression and serious mental illness following diagnosis of COVID-19 in the pre-vaccine availability, vaccinated and unvaccinated cohorts, by sex. Events on the day of COVID-19 diagnosis (day 0) were excluded
eFigure 8. Maximally adjusted hazard ratios and 95% CIs for depression and serious mental illness following diagnosis of COVID-19 in the pre-vaccine availability, vaccinated and unvaccinated cohorts, by ethnicity. Events on the day of COVID-19 diagnosis (day 0) were excluded
eFigure 9. Absolute excess risk up to 28 weeks for depression and serious mental illness following diagnosis of COVID-19 in the pre-vaccine availability, vaccinated and unvaccinated cohorts

This supplemental material has been provided by the authors to give readers additional information about their work.

© 2024 Walker VM et al. *JAMA Psychiatry.*

SUPPLEMENTARY METHODS

Eligibility criteria

People eligible for each cohort were registered with an English GP for at least six months before baseline and were alive with a known age between 18 and 110 years, sex, deprivation, and region at baseline. People were excluded from each cohort if they had any record of SARS-CoV-2 infection before baseline. In the vaccinated cohort, people were excluded if they received a vaccination before the start of the vaccine rollout on December 8th 2020 (indicating an error or participation in a randomized trial); their second dose was dated before their first dose vaccination (contradictory vaccine record); their second dose was less than three weeks after their first dose; or they received mixed vaccine brands before this was permitted on May 7th 2021. In the unvaccinated cohort, people were excluded if they had any record of a COVID-19 vaccination before June 1st 2021. Vaccine eligibility was defined using the Joint Committee on Vaccination and Immunisation (JCVI) groupings. People who could not be assigned to a JCVI group were excluded.

Absolute excess risk

The absolute excess risk analysis was performed for each outcome in each cohort using the hazard ratio from the main analysis. To compare the outcomes across the cohorts, each of which have different lengths of follow-up, we calculated the absolute excess risk at 28 weeks. We accounted for age and sex in the analysis by using event counts and person days from eight subgroups defined by age and sex. They were females, aged 18-39; females, aged 40-59; females, aged 60-79; females, aged 80-110; males, aged 18-39; males, aged 40-59; males, aged 60-79; and males, aged 80-110). For each group, we calculated the average daily incidence of the outcome in the unexposed and the cumulative risk over time. We then used the relevant hazard ratio for that day (e.g., days 0 to 27 will have the coefficient for the term 'days0_28', while days 28 to 196 will have the coefficient for the term 'days28_197') to predict the expected cumulative survival in the exposed. Finally, we calculated the daily excess risk as the difference in cumulative survival for the unexposed and the expected cumulative survival in the exposed. The overall absolute excess risk was estimated using a weighted sum of the excess risks in each group, weighted by the proportions of individuals in age and sex strata in the pre-vaccine availability cohort.

Information governance

NHS England is the data controller of the NHS England OpenSAFELY COVID-19 Service; TPP is the data processor; all study authors using OpenSAFELY have the approval of NHS England. This implementation of OpenSAFELY is hosted within the TPP environment which is accredited to the ISO 27001 information security standard and is NHS IG Toolkit compliant.

Patient data has been pseudonymised for analysis and linkage using industry standard cryptographic hashing techniques; all pseudonymised datasets transmitted for linkage onto OpenSAFELY are encrypted; access to the NHS England OpenSAFELY COVID-19 service is via a virtual private network (VPN) connection; the researchers hold contracts with NHS England and only access the platform to initiate database queries and statistical models; all database activity is logged; only aggregate statistical outputs leave the platform environment following best practice for anonymisation of results such as statistical disclosure control for low cell counts.

The service adheres to the obligations of the UK General Data Protection Regulation (UK GDPR) and the Data Protection Act 2018. The service previously operated under notices initially issued in February 2020 by the the Secretary of State under Regulation 3(4) of the Health Service (Control of Patient Information) Regulations 2002 (COPI Regulations), which required organisations to process confidential patient information for COVID-19 purposes; this set aside the requirement for patient consent. As of 1 July 2023, the Secretary of State has requested that NHS England continue to operate the Service under the COVID-19 Directions 2020. In some cases of data sharing, the common law duty of confidence is met using, for example, patient consent or support from the Health Research Authority Confidentiality Advisory Group.

© 2024 Walker VM et al. *JAMA Psychiatry.*

Taken together, these provide the legal bases to link patient datasets using the service. GP practices, which provide access to the primary care data, are required to share relevant health information to support the public health response to the pandemic and have been informed of how the service operates.

This study was approved by the Health Research Authority [REC reference 22/PR/0095] and by the University of Bristol's Faculty of Health Sciences Ethics Committee [reference 117269].

© 2024 Walker VM et al. *JAMA Psychiatry.*

SUPPLEMENTARY TABLES

eTable 1: Summary of covariate definitions.

Covariate	Type	Definition
Age	Continuous	Modelled as age in years using a restricted cubic spline with three knots at the 10th, 50th and 90th percentiles
Sex	Categorical	Male, Female
Ethnicity	Categorical	1: White<br>2: Mixed<br>3: South Asian<br>4: Black<br>5: Other<br>Missing
Deprivation	Categorical	10 categories from Index of Multiple Deprivation 2019
Region	Categorical	East of England<br>London<br>Midlands<br>North East and Yorkshire<br>North West<br>South East<br>South West<br>Scotland<br>Wales
Smoking status	Categorical	E: Ever smoker<br>M: Missing<br>N: Never smoker<br>S: Current smoker
Care home status	Binary	1 if care home resident; 0 otherwise
Consultation rate	Continuous	Number of GP consultations in 2019
Health care worker	Binary	1 if healthcare worker; 0 otherwise
Dementia	Binary	1 if diagnosis present; 0 otherwise
Liver disease	Binary	1 if diagnosis present; 0 otherwise
Chronic kidney disease	Binary	1 if diagnosis present; 0 otherwise
Cancer	Categorical	1 if diagnosis present; 0 otherwise
Hypertension	Binary	1 if diagnosis present; 0 otherwise
Diabetes	Binary	1 if diagnosis present; 0 otherwise

© 2024 Walker VM et al. *JAMA Psychiatry.*

Obesity	Binary	1 if BMI>=30 or coded diagnosis for obesity; 0 otherwise
Chronic obstructive pulmonary disease	Binary	1 if diagnosis present; 0 otherwise
Acute myocardial infarction	Binary	1 if diagnosis present; 0 otherwise
Ischaemic stroke	Binary	1 if diagnosis present; 0 otherwise
Recent episode of depression	Binary	1 if present ≤6 months prior to index date; 0 otherwise
History of depression	Binary	1 if present >6 months prior to index data; 0 otherwise
Recent episode of anxiety	Binary	1 if present ≤6 months prior to index date; 0 otherwise
History of anxiety	Binary	1 if present >6 months prior to index date; 0 otherwise
Recent diagnosis of an eating disorder	Binary	1 if present ≤6 months prior to index date; 0 otherwise
History of eating disorders	Binary	1 if present >6 months prior to index date; 0 otherwise
Recent report of a serious mental illness	Binary	1 if present ≤6 months prior to index date; 0 otherwise
History of serious mental illness	Binary	1 if present >6 months prior to index date; 0 otherwise
Recent report of self-harm	Binary	1 if present ≤6 months prior to index date; 0 otherwise
History of self-harm	Binary	1 if present >6 months prior to index date; 0 otherwise

© 2024 Walker VM et al. *JAMA Psychiatry.*

eTable 2: Summary of cohort characteristics.

Characteristic	Pre-vaccine availability cohort	Vaccinated cohort	Unvaccinated cohort
Start date	01/01/2020, which is the approximate start date of the pandemic in the UK.	01/06/2021, which is the date that the delta variant was thought to be ubiquitous in England.	01/06/2021, which is the date that the delta variant was thought to be ubiquitous in England.
End date - exposure	18/06/2021, which is the date when the Joint Committee for Vaccination and Immunisation (JCVI) phase 2, group 12 (all adults aged 18 years and older) become eligible for a COVID-19 vaccination.	14/12/2021, which is the day that the UK Health Security Agency stated that over half of English cases they sampled have S Gene Target Failure, meaning they were likely Omicron.*	14/12/2021, which is the day that the UK Health Security Agency stated that over half of English cases they sampled have S Gene Target Failure, meaning they were likely Omicron.*
End date - outcome	14/12/2021, which is the day that the UK Health Security Agency stated that over half of English cases they sampled have S Gene Target Failure, meaning they were likely Omicron.*	14/12/2021, which is the day that the UK Health Security Agency stated that over half of English cases they sampled have S Gene Target Failure, meaning they were likely Omicron.*	14/12/2021, which is the day that the UK Health Security Agency stated that over half of English cases they sampled have S Gene Target Failure, meaning they were likely Omicron.*
Exclusion criteria	Patients will be excluded if they meet any of the following criteria:<br>COVID-19 diagnosis recorded prior to their index date	Patients will be excluded if they meet any of the following criteria:<br>COVID-19 diagnosis recorded prior to their index date [Note: these individuals are required for a sensitivity analysis and so should not be removed at the data extraction stage]<br>They do not have a record of two vaccination doses prior to the study end date<br>They received a vaccination prior to 08-12-2020 (i.e., the start of the vaccination program)<br>They received a second dose vaccination before their first dose vaccination<br>They received a second dose vaccination less than three weeks after their first dose<br>They received mixed vaccine products before 07-05-2021	Patients will be excluded if they meet any of the following criteria:<br>COVID-19 diagnosis recorded prior to their index date [Note: these individuals are required for a sensitivity analysis and so should not be removed at the data extraction stage]<br>They have a record of one or more vaccination doses prior to their index date<br>They could not be assigned to a vaccination group as defined by the Joint Committee on Vaccination and Immunisation (JCVI)
Follow-up start	Study start date.	Follow-up will start at the latest of the following dates (i.e., an individual's index date): Two	Follow-up will start at the latest of the following dates (i.e., an individual's index date):

© 2024 Walker VM et al. *JAMA Psychiatry.*

		weeks after their second vaccination; Study start date.	12 weeks after they became eligible for vaccination; Study start date.
Follow-up end for exposure	Follow-up will end at the earliest of the following dates: Death; Outcome event; Study end date exposure; Deregistration date; Vaccination; Date when eligible for vaccination according to JCVI priority groupings.	Follow-up will end at the earliest of the following dates: Death; Outcome event; Study end date exposure; Deregistration date.	Follow-up will end at the earliest of the following dates: Death; Outcome event; Study end date exposure; Deregistration date; Vaccination.
Follow-up end for outcomes	Follow-up will end at the earliest of the following dates: Death; Outcome event; Study end date outcome; Deregistration date.	Follow-up will end at the earliest of the following dates: Death; Outcome event; Study end date outcome; Deregistration date.	Follow-up will end at the earliest of the following dates: Death; Outcome event; Study end date outcome; Deregistration date.
Cox regression time periods	[0,28), [28,197), [197, 365), [365,714)	[0,28), [28,197)	[0,28), [28,197)
* UK Health Security Agency. (2021). *Omicron daily overview: 17 December 2021*. Retrieved 26 April 2024, from https://assets.publishing.service.gov.uk/government/uploads/system/uploads/attachment_data/file/1042100/20211217_OS_Daily_Omicron_Overview.pdf			

© 2024 Walker VM et al. *JAMA Psychiatry.*

eTable 3: Medical history of people in the pre-vaccine availability, vaccinated and unvaccinated cohorts.

Characteristic		Pre-vaccine availability cohort		Vaccinated cohort		Unvaccinated cohort	
		N (%)	COVID-19 diagnoses	N (%)	COVID-19 diagnoses	N (%)	COVID-19 diagnoses
All		18648606	1012335	14035286	866469	3242215	149745
GP visits in 2019	0	5373888 (28.8%)	228831	3547960 (25.3%)	204849	1779310 (54.9%)	47847
	1-5	6665801 (35.7%)	388053	5246375 (37.4%)	342147	866408 (26.7%)	55209
	6+	6608917 (35.4%)	395457	5240951 (37.3%)	319473	596497 (18.4%)	46683
History of depression	None	13329046 (71.5%)	705051	9725543 (69.3%)	580377	2516902 (77.6%)	101217
	More than six months ago	4845111 (26%)	274557	3998314 (28.5%)	264477	665771 (20.5%)	44055
	Within six months	474449 (2.5%)	32727	311429 (2.2%)	21615	59542 (1.8%)	4473
History of eating disorders	None	18460334 (99%)	1000851	13884963 (98.9%)	855663	3211400 (99%)	147579
	More than six months ago	182150 (1%)	11073	144985 (1%)	10455	29644 (0.9%)	2079
	Within six months	6122 (0%)	417	5338 (0%)	345	1171 (0%)	87
History of general anxiety	None	14789916 (79.3%)	786363	10836194 (77.2%)	651357	2674285 (82.5%)	111057
	More than six months ago	3539886 (19%)	203643	2981611 (21.2%)	199599	521337 (16.1%)	35013
	Within six months	318804 (1.7%)	22323	217481 (1.5%)	15513	46593 (1.4%)	3675
History of self-harm	None	17836869 (95.6%)	965187	13435372 (95.7%)	828939	3076328 (94.9%)	139755
	More than six months ago	784264 (4.2%)	45219	583303 (4.2%)	36531	159352 (4.9%)	9621
	Within six months	27473 (0.1%)	1923	16611 (0.1%)	999	6535 (0.2%)	369
History of serious mental illness	None	16012454 (85.9%)	860733	11920615 (84.9%)	728301	2854694 (88%)	125151
	More than six months ago	2497561 (13.4%)	141993	2032905 (14.5%)	132813	368079 (11.4%)	23319
	Within six months	138591 (0.7%)	9609	81766 (0.6%)	5355	19442 (0.6%)	1275
Medical history	Acute myocardial infarction	470120 (2.5%)	23355	426477 (3%)	16857	21956 (0.7%)	1143
	Cancer	5477559 (29.4%)	335295	4763859 (33.9%)	362139	613040 (18.9%)	50175
	Chronic kidney disease	1182309 (6.3%)	58005	1072844 (7.6%)	38955	48350 (1.5%)	2721
	COPD	612345 (3.3%)	29205	542087 (3.9%)	21099	29288 (0.9%)	1431
	Dementia	232174 (1.2%)	23619	177258 (1.3%)	6525	6240 (0.2%)	345
	Diabetes	1657130 (8.9%)	97707	1489500 (10.6%)	79797	124349 (3.8%)	8067
	Hypertension	573598 (3.1%)	64965	487872 (3.5%)	39045	20415 (0.6%)	1947
	Liver disease	6389682 (34.3%)	320835	5578402 (39.7%)	289053	493021 (15.2%)	31749
	Obesity	135394 (0.7%)	7137	117507 (0.8%)	5793	16479 (0.5%)	741
	Stroke	4568196 (24.5%)	287343	3864178 (27.5%)	264789	483135 (14.9%)	33657

COPD: chronic obstructive pulmonary disease.

eTable 4: Mental illness events following diagnosis of COVID-19 in the pre-vaccine availability, vaccinated and unvaccinated cohorts, by prior history of the outcome.

Outcome	Prior history of the outcome	Pre-vaccine availability cohort N=18,648,606		Vaccinated cohort N=14,035,286		Unvaccinated cohort N=3,242,215	
		Event/person-years	Incidence rate	Event/person-years	Incidence rate	Event/person-years	Incidence rate
Depression	None	17415/684144	2546	2433/106288	2289	549/17995	3051
	Within six months	5127/19141	26785	1665/3299	50475	489/665	73485
	More than six months ago	28287/235464	12013	7035/49036	14347	1869/8010	23334
Serious mental illness	None	8109/851371	952	1113/135243	823	255/22601	1128
	Within six months	969/6973	13896	279/893	31256	99/218	45513
	More than six months ago	5979/134054	4460	1185/25568	4635	381/4443	8574

eTable 5: Maximally adjusted hazard ratios and 95% CIs for mental illness events following diagnosis of COVID-19 in the pre-vaccine availability, vaccinated and unvaccinated cohorts.

Outcome	Time since COVID-19	Pre-vaccine availability cohort	Vaccinated cohort	Unvaccinated cohort
Depression	Day 0	33.2 (32.1-34.3)	8.54 (8.07-9.04)	19.0 (17.4-20.7)
	1-4 weeks	1.93 (1.88-1.98)	1.16 (1.12-1.20)	1.79 (1.68-1.90)
	5-28 weeks	1.25 (1.23-1.27)	1.11 (1.08-1.14)	1.28 (1.21-1.36)
	29-52 weeks	1.16 (1.14-1.18)	-	-
	53-102 weeks	1.17 (1.14-1.21)	-	-
Serious mental illness	Day 0	30.1 (28.3-32.0)	7.68 (6.82-8.65)	19.5 (16.6-22.9)
	1-4 weeks	1.49 (1.41-1.57)	0.91 (0.85-0.98)	1.45 (1.27-1.65)
	5-28 weeks	1.20 (1.17-1.23)	1.05 (1.00-1.11)	1.14 (1.02-1.27)
	29-52 weeks	1.15 (1.12-1.18)	-	-
	53-102 weeks	1.14 (1.08-1.21)	-	-
General anxiety	Day 0	34.0 (32.8-35.3)	8.51 (7.95-9.12)	25.2 (23.2-27.3)
	1-4 weeks	2.18 (2.12-2.25)	1.25 (1.21-1.30)	2.28 (2.14-2.43)
	5-28 weeks	1.26 (1.24-1.28)	1.12 (1.09-1.16)	1.36 (1.27-1.44)
	29-52 weeks	1.14 (1.12-1.16)	-	-
	53-102 weeks	1.15 (1.11-1.19)	-	-
Post-traumatic stress disorder	Day 0	40.6 (34.1-48.4)	12.6 (9.44-16.8)	30.3 (22.5-40.8)
	1-4 weeks	1.84 (1.57-2.17)	1.06 (0.85-1.31)	1.45 (1.08-1.94)
	5-28 weeks	1.15 (1.06-1.25)	1.04 (0.89-1.22)	1.06 (0.82-1.38)
	29-52 weeks	1.09 (1.00-1.19)	-	-
	53-102 weeks	1.19 (1.01-1.41)	-	-
Eating disorders	Day 0	25.5 (19.6-33.2)	5.99 (3.51-10.2)	20.7 (11.6-36.9)
	1-4 weeks	1.86 (1.52-2.28)	0.99 (0.73-1.35)	1.57 (0.98-2.53)
	5-28 weeks	1.20 (1.06-1.36)	1.00 (0.79-1.26)	1.14 (0.73-1.78)
	29-52 weeks	1.04 (0.90-1.21)	-	-
	53-102 weeks	1.13 (0.85-1.49)	-	-
Addiction	Day 0	66.1 (56.9-76.7)	15.5 (10.9-22.0)	42.6 (33.1-54.8)
	1-4 weeks	1.49 (1.22-1.81)	0.87 (0.64-1.20)	1.01 (0.71-1.43)
	5-28 weeks	0.95 (0.86-1.05)	0.66 (0.50-0.86)	0.77 (0.57-1.04)
	29-52 weeks	0.99 (0.89-1.11)	-	-
	53-102 weeks	0.97 (0.77-1.22)	-	-
Self-harm	Day 0	23.3 (20.2-26.9)	10.7 (8.49-13.6)	12.6 (8.75-18.3)
	1-4 weeks	1.22 (1.08-1.38)	1.00 (0.85-1.18)	1.12 (0.85-1.47)
	5-28 weeks	1.16 (1.10-1.23)	1.15 (1.03-1.29)	0.95 (0.76-1.19)
	29-52 weeks	1.16 (1.10-1.24)	-	-
	53-102 weeks	1.20 (1.06-1.35)	-	-
Suicide	Day 0	7.82 (1.98-30.9)	†	†
	1-4 weeks	2.69 (1.69-4.29)	†	†
	5-28 weeks	0.87 (0.63-1.21)	†	†
	29-52 weeks	0.81 (0.56-1.16)	-	-
	53-102 weeks	1.05 (0.56-1.96)	-	-

† Insufficient events for estimation

eTable 6: Maximally adjusted hazard ratios and 95% CIs for mental illness events following hospitalised COVID-19 in the pre-vaccine availability, vaccinated and unvaccinated cohorts.

Outcome	Time since COVID-19	Pre-vaccine availability cohort	Vaccinated cohort	Unvaccinated cohort
Depression	Day 0	263 (251-275)	129 (118-142)	267 (244-293)
	1-4 weeks	16.3 (15.6-17.0)	12.9 (12.0-14.0)	15.6 (13.9-17.4)
	5-28 weeks	1.83 (1.72-1.93)	1.56 (1.33-1.83)	1.93 (1.60-2.33)
	29-52 weeks	1.45 (1.35-1.55)	-	-
	53-102 weeks	1.36 (1.23-1.51)	-	-
Serious mental illness	Day 0	230 (211-252)	102 (82.2-127)	139 (109-178)
	1-4 weeks	9.71 (8.80-10.7)	6.52 (5.36-7.93)	8.75 (7.01-10.9)
	5-28 weeks	1.58 (1.41-1.75)	0.88 (0.60-1.30)	0.96 (0.61-1.51)
	29-52 weeks	1.37 (1.21-1.55)	-	-
	53-102 weeks	1.07 (0.87-1.33)	-	-
General anxiety	Day 0	307 (291-324)	150 (134-168)	274 (243-308)
	1-4 weeks	21.1 (20.1-22.1)	16.9 (15.6-18.4)	26.9 (24.3-29.7)
	5-28 weeks	1.97 (1.84-2.10)	1.65 (1.37-1.99)	3.00 (2.52-3.55)
	29-52 weeks	1.44 (1.32-1.56)	-	-
	53-102 weeks	1.23 (1.09-1.39)	-	-
Post-traumatic stress disorder	Day 0	405 (312-527)	285 (181-447)	345 (230-518)
	1-4 weeks	20.1 (15.8-25.6)	27.3 (20.3-36.6)	13.3 (8.00-22.2)
	5-28 weeks	4.19 (3.38-5.19)	2.01 (0.91-4.42)	2.49 (1.26-4.93)
	29-52 weeks	2.57 (1.94-3.40)	-	-
	53-102 weeks	2.11 (1.31-3.41)	-	-
Eating disorders	Day 0	164 (92.9-291)	†	†
	1-4 weeks	7.94 (4.47-14.1)	†	†
	5-28 weeks	1.46 (0.83-2.58)	†	†
	29-52 weeks	1.18 (0.62-2.27)	-	-
	53-102 weeks	1.87 (0.92-3.77)	-	-
Addiction	Day 0	311 (231-418)	†	322 (223-465)
	1-4 weeks	10.2 (7.27-14.2)	†	7.70 (4.61-12.9)
	5-28 weeks	1.85 (1.32-2.58)	†	0.74 (0.24-2.30)
	29-52 weeks	2.00 (1.38-2.90)	-	-
	53-102 weeks	1.80 (0.99-3.25)	-	-
Self-harm	Day 0	80.1 (55.6-115)	†	†
	1-4 weeks	3.78 (2.68-5.34)	†	†
	5-28 weeks	1.92 (1.56-2.36)	†	†
	29-52 weeks	1.64 (1.27-2.11)	-	-
	53-102 weeks	1.95 (1.41-2.70)	-	-
Suicide	Day 0	†	†	†
	1-4 weeks	†	†	†
	5-28 weeks	†	†	†
	29-52 weeks	†	-	-
	53-102 weeks	†	-	-

† Insufficient events for estimation

eTable 7: Maximally adjusted hazard ratios and 95% Cis for mental illness events following non-hospitalised COVID-19 in the pre-vaccine availability, vaccinated and unvaccinated cohorts.

Outcome	Time since COVID-19	Pre-vaccine availability cohort	Vaccinated cohort	Unvaccinated cohort
Depression	Day 0	18.0 (17.3-18.9)	5.42 (5.04-5.82)	7.68 (6.69-8.82)
	1-4 weeks	1.22 (1.18-1.27)	0.92 (0.88-0.95)	1.11 (1.02-1.20)
	5-28 weeks	1.22 (1.21-1.24)	1.10 (1.07-1.13)	1.26 (1.19-1.34)
	29-52 weeks	1.15 (1.13-1.17)	-	-
	53-102 weeks	1.16 (1.12-1.19)	-	-
Serious mental illness	Day 0	17.5 (16.1-19.0)	5.46 (4.73-6.29)	11.7 (9.45-14.5)
	1-4 weeks	1.05 (0.98-1.12)	0.79 (0.73-0.86)	1.00 (0.85-1.17)
	5-28 weeks	1.18 (1.15-1.22)	1.05 (1.00-1.11)	1.14 (1.02-1.28)
	29-52 weeks	1.14 (1.11-1.17)	-	-
	53-102 weeks	1.15 (1.09-1.22)	-	-
General anxiety	Day 0	19.0 (18.1-20.0)	5.51 (5.05-6.00)	9.33 (8.09-10.7)
	1-4 weeks	1.38 (1.33-1.43)	0.97 (0.93-1.01)	1.25 (1.14-1.36)
	5-28 weeks	1.23 (1.21-1.25)	1.11 (1.08-1.15)	1.30 (1.22-1.39)
	29-52 weeks	1.13 (1.11-1.15)	-	-
	53-102 weeks	1.14 (1.10-1.18)	-	-
Post-traumatic stress disorder	Day 0	22.0 (17.4-27.9)	7.03 (4.80-10.3)	13.6 (8.70-21.2)
	1-4 weeks	0.96 (0.77-1.20)	0.62 (0.47-0.82)	0.66 (0.43-1.02)
	5-28 weeks	0.99 (0.91-1.08)	1.02 (0.87-1.19)	0.95 (0.72-1.26)
	29-52 weeks	1.00 (0.91-1.09)	-	-
	53-102 weeks	1.07 (0.90-1.28)	-	-
Eating disorders	Day 0	17.6 (12.5-24.8)	5.31 (2.99-9.44)	†
	1-4 weeks	1.53 (1.23-1.92)	0.85 (0.61-1.19)	†
	5-28 weeks	1.23 (1.10-1.38)	1.06 (0.84-1.33)	†
	29-52 weeks	1.08 (0.95-1.23)	-	-
	53-102 weeks	1.16 (0.90-1.48)	-	-
Addiction	Day 0	49.6 (41.5-59.4)	12.5 (8.36-18.7)	22.5 (15.7-32.0)
	1-4 weeks	1.01 (0.79-1.29)	0.70 (0.49-1.01)	0.52 (0.31-0.86)
	5-28 weeks	0.91 (0.82-1.01)	0.66 (0.50-0.86)	0.78 (0.57-1.07)
	29-52 weeks	0.95 (0.85-1.07)	-	-
	53-102 weeks	0.89 (0.69-1.14)	-	-
Self-harm	Day 0	19.9 (17.0-23.4)	10.1 (7.92-12.9)	10.8 (7.11-16.3)
	1-4 weeks	1.11 (0.97-1.26)	0.93 (0.78-1.10)	1.08 (0.81-1.44)
	5-28 weeks	1.13 (1.07-1.19)	1.11 (0.99-1.24)	0.95 (0.76-1.21)
	29-52 weeks	1.14 (1.07-1.21)	-	-
	53-102 weeks	1.11 (0.97-1.26)	-	-
Suicide	Day 0	8.52 (2.15-33.7)	†	†
	1-4 weeks	2.42 (1.46-4.04)	†	†
	5-28 weeks	0.86 (0.62-1.21)	†	†
	29-52 weeks	0.80 (0.55-1.17)	-	-
	53-102 weeks	0.94 (0.47-1.89)	-	-

† Insufficient events for estimation

eTable 8: Maximally adjusted hazard ratios and 95% CIs for mental illness events following diagnosis of COVID-19 in the pre-vaccine availability, vaccinated and unvaccinated cohorts, in people with no prior history of the outcome.

Outcome	Time since COVID-19	Pre-vaccine availability cohort	Vaccinated cohort	Unvaccinated cohort
Depression	Day 0	18.0 (16.7-19.5)	5.83 (5.03-6.76)	11.6 (9.09-14.8)
	1-4 weeks	1.59 (1.51-1.68)	1.11 (1.03-1.20)	1.47 (1.26-1.71)
	5-28 weeks	1.41 (1.38-1.44)	1.26 (1.20-1.33)	1.53 (1.36-1.71)
	29-52 weeks	1.23 (1.20-1.27)	-	-
	53-102 weeks	1.31 (1.25-1.38)	-	-
Serious mental illness	Day 0	11.7 (10.3-13.3)	4.11 (3.19-5.30)	9.28 (6.55-13.1)
	1-4 weeks	1.19 (1.09-1.29)	0.95 (0.84-1.06)	1.21 (0.97-1.51)
	5-28 weeks	1.32 (1.27-1.36)	1.20 (1.12-1.30)	1.25 (1.06-1.47)
	29-52 weeks	1.24 (1.20-1.29)	-	-
	53-102 weeks	1.23 (1.14-1.32)	-	-

eTable 9: Maximally adjusted hazard ratios and 95% CIs for mental illness events following diagnosis of COVID-19 in the pre-vaccine availability, vaccinated and unvaccinated cohorts, in people with prior history of the outcome, more than six months ago.

Outcome	Time since COVID-19	Pre-vaccine availability cohort	Vaccinated cohort	Unvaccinated cohort
Depression	Day 0	39.8 (38.2-41.4)	10.8 (10.2-11.5)	24.2 (21.9-26.8)
	1-4 weeks	2.16 (2.09-2.24)	1.23 (1.18-1.28)	2.06 (1.91-2.22)
	5-28 weeks	1.17 (1.15-1.19)	1.11 (1.07-1.15)	1.28 (1.20-1.38)
	29-52 weeks	1.12 (1.09-1.14)	-	-
	53-102 weeks	1.07 (1.03-1.12)	-	-
Serious mental illness	Day 0	46.0 (42.6-49.7)	11.7 (10.3-13.4)	27.8 (22.9-33.9)
	1-4 weeks	1.80 (1.66-1.95)	0.90 (0.80-1.00)	1.62 (1.36-1.93)
	5-28 weeks	1.08 (1.04-1.13)	1.00 (0.92-1.07)	1.01 (0.86-1.18)
	29-52 weeks	1.07 (1.02-1.12)	-	-
	53-102 weeks	1.09 (1.00-1.19)	-	-

eTable 10: Maximally adjusted hazard ratios and 95% CIs for mental illness events following diagnosis of COVID-19 in the pre-vaccine availability, vaccinated and unvaccinated cohorts, in people with prior history of the outcome, within six months.

Outcome	Time since COVID-19	Pre-vaccine availability cohort	Vaccinated cohort	Unvaccinated cohort
Depression	Day 0	40.4 (37.5-43.4)	9.46 (8.45-10.6)	12.3 (10.0-15.1)
	1-4 weeks	1.67 (1.54-1.81)	0.99 (0.91-1.08)	1.33 (1.15-1.53)
	5-28 weeks	1.07 (1.03-1.12)	0.92 (0.86-0.99)	1.02 (0.89-1.17)
	29-52 weeks	1.01 (0.96-1.07)	-	-
	53-102 weeks	1.12 (1.01-1.24)	-	-
Serious mental illness	Day 0	65.8 (57.7-75.1)	13.0 (10.2-16.5)	20.1 (13.6-29.9)
	1-4 weeks	1.90 (1.60-2.27)	1.03 (0.84-1.27)	1.33 (0.94-1.89)
	5-28 weeks	1.01 (0.91-1.12)	0.86 (0.72-1.02)	1.12 (0.83-1.49)
	29-52 weeks	0.87 (0.76-0.99)	-	-
	53-102 weeks	1.04 (0.82-1.32)	-	-

eTable 11: Maximally adjusted hazard ratios and 95% CIs for mental illness events following diagnosis of COVID-19 in the pre-vaccine availability, vaccinated and unvaccinated cohorts, in people with history of COVID-19.

Outcome	Time since COVID-19	Vaccinated cohort	Unvaccinated cohort
Depression	Day 0	38.7 (31.5-47.4)	47.9 (32.8-70.0)
	1-4 weeks	1.25 (0.95-1.66)	2.42 (1.63-3.61)
	5-28 weeks	1.12 (0.93-1.33)	1.60 (1.14-2.25)
Serious mental illness	Day 0	46.3 (31.4-68.2)	†
	1-4 weeks	0.67 (0.32-1.42)	†
	5-28 weeks	0.90 (0.60-1.34)	†

† Insufficient events for estimation

eTable 12: Maximally adjusted hazard ratios and 95% CIs for mental illness events following diagnosis of COVID-19 in the pre-vaccine availability, vaccinated and unvaccinated cohorts for age group 18-39.

Outcome	Time since COVID-19	Pre-vaccine availability cohort	Vaccinated cohort	Unvaccinated cohort
Depression	Day 0	6.18 (5.55-6.88)	2.14 (1.77-2.59)	6.98 (5.90-8.25)
	1-4 weeks	1.07 (1.02-1.13)	0.88 (0.82-0.94)	1.27 (1.16-1.39)
	5-28 weeks	1.19 (1.17-1.22)	1.06 (1.01-1.10)	1.22 (1.13-1.31)
	29-52 weeks	1.16 (1.13-1.19)	-	-
	53-102 weeks	1.20 (1.14-1.26)	-	-
Serious mental illness	Day 0	6.41 (5.34-7.69)	1.89 (1.28-2.77)	8.36 (6.22-11.2)
	1-4 weeks	0.99 (0.90-1.09)	0.81 (0.71-0.92)	1.03 (0.86-1.25)
	5-28 weeks	1.13 (1.09-1.17)	1.06 (0.97-1.15)	1.14 (0.99-1.32)
	29-52 weeks	1.13 (1.09-1.18)	-	-
	53-102 weeks	1.07 (0.98-1.17)	-	-

eTable 13: Maximally adjusted hazard ratios and 95% CIs for mental illness events following diagnosis of COVID-19 in the pre-vaccine availability, vaccinated and unvaccinated cohorts for age group 40-59.

Outcome	Time since COVID-19	Pre-vaccine availability cohort	Vaccinated cohort	Unvaccinated cohort
Depression	Day 0	24.7 (23.2-26.4)	4.51 (4.01-5.08)	29.5 (25.7-33.9)
	1-4 weeks	2.10 (2.01-2.20)	1.11 (1.05-1.17)	2.38 (2.14-2.65)
	5-28 weeks	1.22 (1.19-1.25)	1.13 (1.09-1.17)	1.36 (1.24-1.50)
	29-52 weeks	1.16 (1.13-1.19)	-	-
	53-102 weeks	1.21 (1.15-1.27)	-	-
Serious mental illness	Day 0	22.9 (20.2-25.9)	4.58 (3.63-5.78)	24.4 (18.6-32.2)
	1-4 weeks	1.41 (1.28-1.56)	0.82 (0.73-0.92)	1.59 (1.26-2.00)
	5-28 weeks	1.12 (1.07-1.17)	1.05 (0.97-1.13)	1.10 (0.91-1.33)
	29-52 weeks	1.09 (1.04-1.15)	-	-
	53-102 weeks	1.22 (1.11-1.35)	-	-

eTable 14: Maximally adjusted hazard ratios and 95% CIs for mental illness events following diagnosis of COVID-19 in the pre-vaccine availability, vaccinated and unvaccinated cohorts for age group 60-79.

Outcome	Time since COVID-19	Pre-vaccine availability cohort	Vaccinated cohort	Unvaccinated cohort
Depression	Day 0	111 (105-117)	23.4 (21.4-25.7)	159 (133-189)
	1-4 weeks	4.45 (4.21-4.70)	1.68 (1.56-1.81)	7.66 (6.35-9.25)
	5-28 weeks	1.46 (1.40-1.52)	1.17 (1.10-1.25)	1.76 (1.34-2.31)
	29-52 weeks	1.19 (1.14-1.24)	-	-
	53-102 weeks	1.11 (1.03-1.20)	-	-
Serious mental illness	Day 0	121 (110-132)	27.1 (22.7-32.3)	117 (82.6-166)
	1-4 weeks	3.36 (2.99-3.78)	1.35 (1.14-1.60)	6.03 (4.20-8.65)
	5-28 weeks	1.56 (1.45-1.68)	1.04 (0.91-1.20)	1.56 (0.95-2.56)
	29-52 weeks	1.31 (1.20-1.42)	-	-
	53-102 weeks	1.08 (0.93-1.26)	-	-

eTable 15: Maximally adjusted hazard ratios and 95% CIs for mental illness events following diagnosis of COVID-19 in the pre-vaccine availability, vaccinated and unvaccinated cohorts for age group 80-110.

Outcome	Time since COVID-19	Pre-vaccine availability cohort	Vaccinated cohort	Unvaccinated cohort
Depression	Day 0	128 (119-138)	71.3 (63.5-80.0)	253 (178-360)
	1-4 weeks	3.18 (2.87-3.52)	2.70 (2.36-3.08)	6.29 (3.65-10.9)
	5-28 weeks	1.40 (1.31-1.51)	1.24 (1.07-1.43)	1.86 (0.87-3.95)
	29-52 weeks	1.11 (1.02-1.21)	-	-
	53-102 weeks	0.90 (0.80-1.01)	-	-
Serious mental illness	Day 0	98.5 (83.7-116)	61.9 (46.9-81.6)	†
	1-4 weeks	3.40 (2.80-4.13)	2.03 (1.45-2.83)	†
	5-28 weeks	1.65 (1.45-1.88)	1.22 (0.89-1.66)	†
	29-52 weeks	1.34 (1.15-1.57)	-	-
	53-102 weeks	1.06 (0.86-1.30)	-	-

† Insufficient events for estimation

eTable 16: Maximally adjusted hazard ratios and 95% CIs for mental illness events following diagnosis of COVID-19 in the pre-vaccine availability, vaccinated and unvaccinated cohorts for women.

Outcome	Time since COVID-19	Pre-vaccine availability cohort	Vaccinated cohort	Unvaccinated cohort
Depression	Day 0	26.7 (25.5-27.9)	7.36 (6.83-7.92)	17.0 (15.3-19.0)
	1-4 weeks	1.78 (1.72-1.84)	1.10 (1.06-1.15)	1.63 (1.51-1.76)
	5-28 weeks	1.24 (1.21-1.26)	1.11 (1.08-1.14)	1.25 (1.17-1.34)
	29-52 weeks	1.15 (1.13-1.17)	-	-
	53-102 weeks	1.16 (1.12-1.21)	-	-
Serious mental illness	Day 0	25.7 (23.7-28.0)	6.26 (5.31-7.38)	15.4 (12.2-19.4)
	1-4 weeks	1.35 (1.26-1.46)	0.87 (0.79-0.96)	1.20 (1.00-1.44)
	5-28 weeks	1.19 (1.15-1.23)	1.09 (1.02-1.16)	1.13 (0.98-1.30)
	29-52 weeks	1.14 (1.10-1.19)	-	-
	53-102 weeks	1.13 (1.06-1.22)	-	-

eTable 17: Maximally adjusted hazard ratios and 95% CIs for mental illness events following diagnosis of COVID-19 in the pre-vaccine availability, vaccinated and unvaccinated cohorts for men.

Outcome	Time since COVID-19	Pre-vaccine availability cohort	Vaccinated cohort	Unvaccinated cohort
Depression	Day 0	46.1 (43.9-48.4)	11.1 (10.2-12.2)	24.3 (21.1-28.0)
	1-4 weeks	2.26 (2.16-2.36)	1.29 (1.22-1.37)	2.19 (1.97-2.43)
	5-28 weeks	1.27 (1.24-1.30)	1.11 (1.06-1.16)	1.38 (1.25-1.53)
	29-52 weeks	1.18 (1.15-1.21)	-	-
	53-102 weeks	1.18 (1.11-1.24)	-	-
Serious mental illness	Day 0	38.4 (35.2-41.9)	10.3 (8.71-12.2)	25.9 (20.7-32.4)
	1-4 weeks	1.71 (1.57-1.86)	0.99 (0.88-1.12)	1.84 (1.53-2.22)
	5-28 weeks	1.23 (1.18-1.28)	1.00 (0.92-1.09)	1.17 (0.98-1.39)
	29-52 weeks	1.17 (1.11-1.22)	-	-
	53-102 weeks	1.16 (1.06-1.28)	-	-

eTable 18: Maximally adjusted hazard ratios and 95% CIs for mental illness events following diagnosis of COVID-19 in the pre-vaccine availability, vaccinated and unvaccinated cohorts for White ethnicity.

Outcome	Time since COVID-19	Pre-vaccine availability cohort	Vaccinated cohort	Unvaccinated cohort
Depression	Day 0	34.4 (33.2-35.6)	8.66 (8.16-9.18)	18.3 (16.7-20.1)
	1-4 weeks	1.93 (1.87-1.99)	1.16 (1.12-1.20)	1.74 (1.63-1.86)
	5-28 weeks	1.24 (1.22-1.26)	1.11 (1.08-1.13)	1.26 (1.18-1.34)
	29-52 weeks	1.15 (1.13-1.17)	-	-
	53-102 weeks	1.17 (1.13-1.21)	-	-
Serious mental illness	Day 0	29.8 (27.9-31.8)	7.67 (6.77-8.69)	17.7 (14.7-21.2)
	1-4 weeks	1.42 (1.34-1.51)	0.92 (0.85-0.99)	1.36 (1.17-1.57)
	5-28 weeks	1.21 (1.17-1.24)	1.05 (1.00-1.11)	1.11 (0.99-1.26)
	29-52 weeks	1.15 (1.11-1.18)	-	-
	53-102 weeks	1.14 (1.08-1.21)	-	-

eTable 19: Maximally adjusted hazard ratios and 95% CIs for mental illness events following diagnosis of COVID-19 in the pre-vaccine availability, vaccinated and unvaccinated cohorts for South Asian ethnicity.

Outcome	Time since COVID-19	Pre-vaccine availability cohort	Vaccinated cohort	Unvaccinated cohort
Depression	Day 0	28.7 (25.1-32.8)	12.2 (9.22-16.2)	35.1 (25.8-47.7)
	1-4 weeks	2.01 (1.81-2.23)	1.14 (0.93-1.39)	2.13 (1.63-2.79)
	5-28 weeks	1.34 (1.27-1.41)	1.16 (1.01-1.34)	1.37 (1.07-1.76)
	29-52 weeks	1.23 (1.16-1.30)	-	-
	53-102 weeks	1.14 (1.02-1.27)	-	-
Serious mental illness	Day 0	35.2 (28.8-42.9)	10.3 (6.08-17.4)	†
	1-4 weeks	1.69 (1.41-2.02)	0.87 (0.59-1.28)	†
	5-28 weeks	1.14 (1.04-1.25)	0.92 (0.71-1.21)	†
	29-52 weeks	1.07 (0.97-1.19)	-	-
	53-102 weeks	0.99 (0.82-1.20)	-	-

† Insufficient events for estimation

eTable 20: Maximally adjusted hazard ratios and 95% CIs for mental illness events following diagnosis of COVID-19 in the pre-vaccine availability, vaccinated and unvaccinated cohorts for Black ethnicity.

Outcome	Time since COVID-19	Pre-vaccine availability cohort	Vaccinated cohort	Unvaccinated cohort
Depression	Day 0	37.9 (29.7-48.4)	8.99 (4.82-16.8)	17.7 (11.0-28.4)
	1-4 weeks	1.95 (1.57-2.42)	1.50 (1.05-2.13)	1.90 (1.37-2.65)
	5-28 weeks	1.10 (0.98-1.24)	1.16 (0.86-1.56)	1.39 (1.06-1.82)
	29-52 weeks	1.23 (1.09-1.38)	-	-
	53-102 weeks	0.89 (0.68-1.15)	-	-
Serious mental illness	Day 0	54.2 (39.8-73.8)	†	†
	1-4 weeks	2.68 (2.02-3.56)	†	†
	5-28 weeks	0.97 (0.81-1.18)	†	†
	29-52 weeks	1.15 (0.95-1.40)	-	-
	53-102 weeks	1.20 (0.83-1.74)	-	-

† Insufficient events for estimation

eTable 21: Maximally adjusted hazard ratios and 95% CIs for mental illness events following diagnosis of COVID-19 in the pre-vaccine availability, vaccinated and unvaccinated cohorts for Other ethnicity.

Outcome	Time since COVID-19	Pre-vaccine availability cohort	Vaccinated cohort	Unvaccinated cohort
Depression	Day 0	35.2 (26.3-47.0)	3.35 (1.26-8.89)	30.4 (16.2-57.0)
	1-4 weeks	2.45 (1.96-3.07)	1.39 (0.99-1.97)	2.79 (1.73-4.53)
	5-28 weeks	1.60 (1.42-1.79)	1.20 (0.91-1.59)	2.27 (1.50-3.43)
	29-52 weeks	1.36 (1.19-1.56)	-	-
	53-102 weeks	1.28 (0.97-1.68)	-	-
Serious mental illness	Day 0	24.8 (14.3-42.9)	†	†
	1-4 weeks	1.48 (0.94-2.33)	†	†
	5-28 weeks	1.55 (1.28-1.87)	†	†
	29-52 weeks	1.49 (1.21-1.84)	-	-
	53-102 weeks	1.00 (0.61-1.64)	-	-

† Insufficient events for estimation

eTable 22: Maximally adjusted hazard ratios and 95% CIs for mental illness events following diagnosis of COVID-19 in the pre-vaccine availability, vaccinated and unvaccinated cohorts for Mixed ethnicity.

Outcome	Time since COVID-19	Pre-vaccine availability cohort	Vaccinated cohort	Unvaccinated cohort
Depression	Day 0	22.0 (15.6-31.0)	4.58 (2.07-10.1)	13.0 (7.12-23.7)
	1-4 weeks	1.52 (1.16-1.98)	0.84 (0.56-1.26)	2.06 (1.47-2.89)
	5-28 weeks	1.21 (1.07-1.37)	0.84 (0.62-1.12)	0.90 (0.61-1.31)
	29-52 weeks	1.09 (0.95-1.25)	-	-
	53-102 weeks	1.50 (1.17-1.91)	-	-
Serious mental illness	Day 0	24.1 (14.2-41.0)	†	†
	1-4 weeks	2.27 (1.60-3.20)	†	†
	5-28 weeks	1.04 (0.84-1.29)	†	†
	29-52 weeks	1.11 (0.89-1.39)	-	-
	53-102 weeks	1.75 (1.20-2.54)	-	-

† Insufficient events for estimation

eTable 23: Excess events per 100,000 people at 28 weeks post-COVID-19 in the pre-vaccine availability, vaccinated and unvaccinated cohorts.

Outcome	Pre-vaccine availability cohort			Vaccinated cohort			Unvaccinated cohort		
	Day zero included	Day zero excluded	Difference	Day zero included	Day zero excluded	Difference	Day zero included	Day zero excluded	Difference
Depression	1030	697	333	449	336	113	1005	799	207
Serious mental illness	234	145	89	53	26	27	208	138	71
General anxiety	826	576	249	366	288	78	1004	801	203
PTSD	26	14	12	8	3	5	29	13	16
Eating disorders	13	9	4	1	0	1	11	8	4
Addiction	21	1	20	-12	-16	4	2	-31	33
Self-harm	40	24	16	29	21	8	6	-5	11
Suicide	1	1	0	†	†	†	†	†	†

† Insufficient events for estimation; PTSD = post-traumatic stress disorder.

EFIGURES

eFigure 1: COVID-19 cases over time

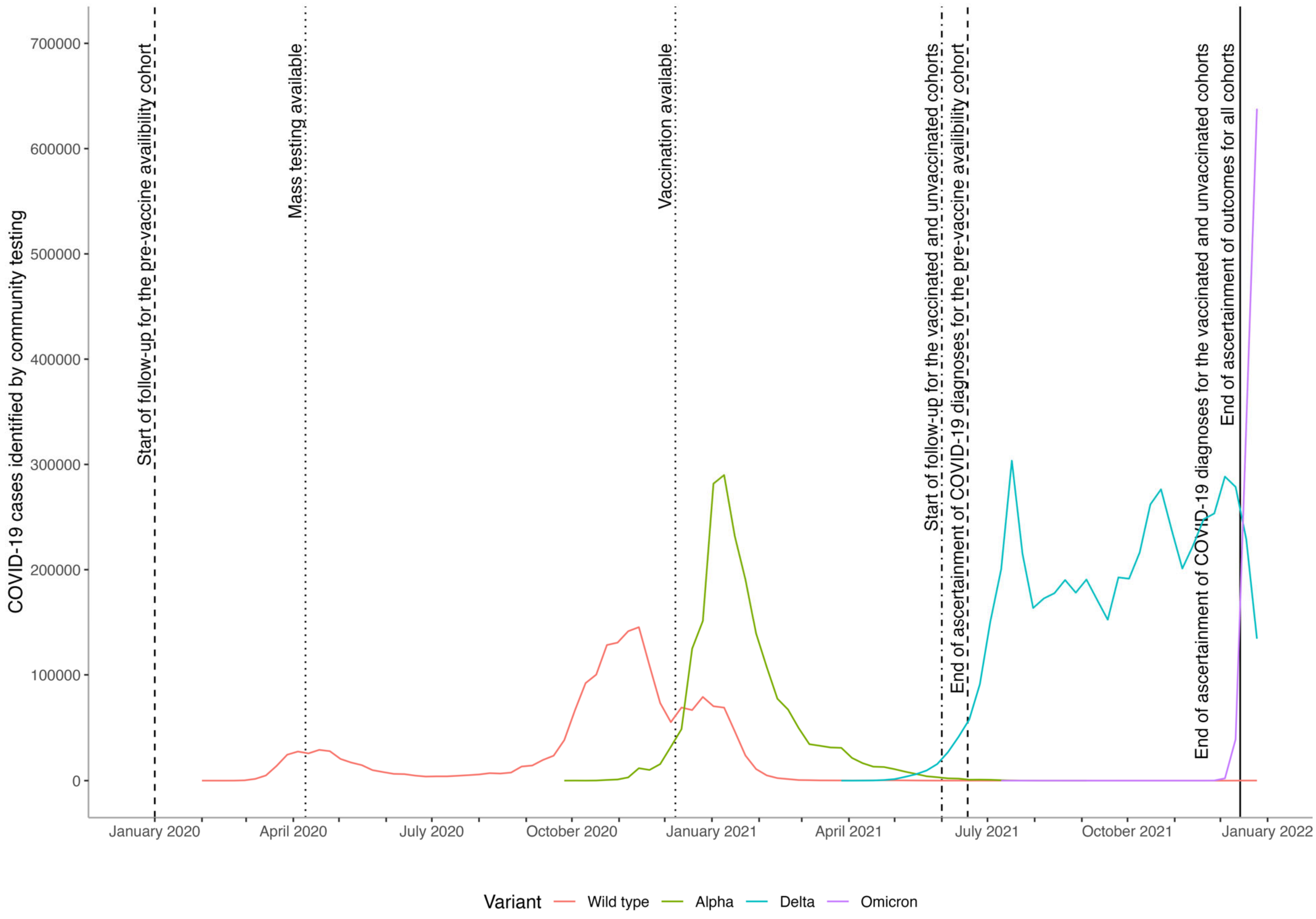

eFigure 2: Diagram showing cohort construction.

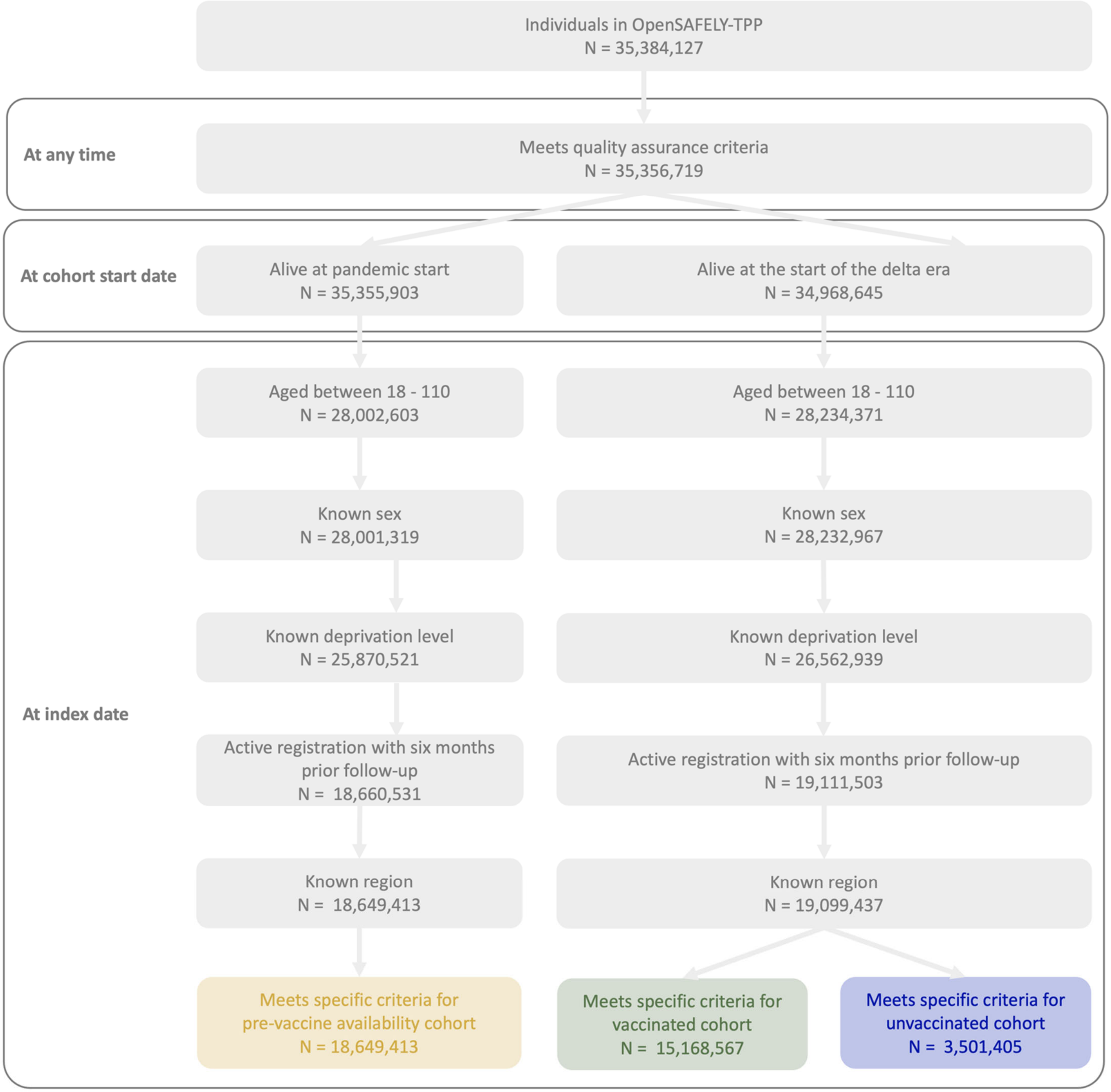

eFigure 3: Venn Diagram showing cohort overlap.

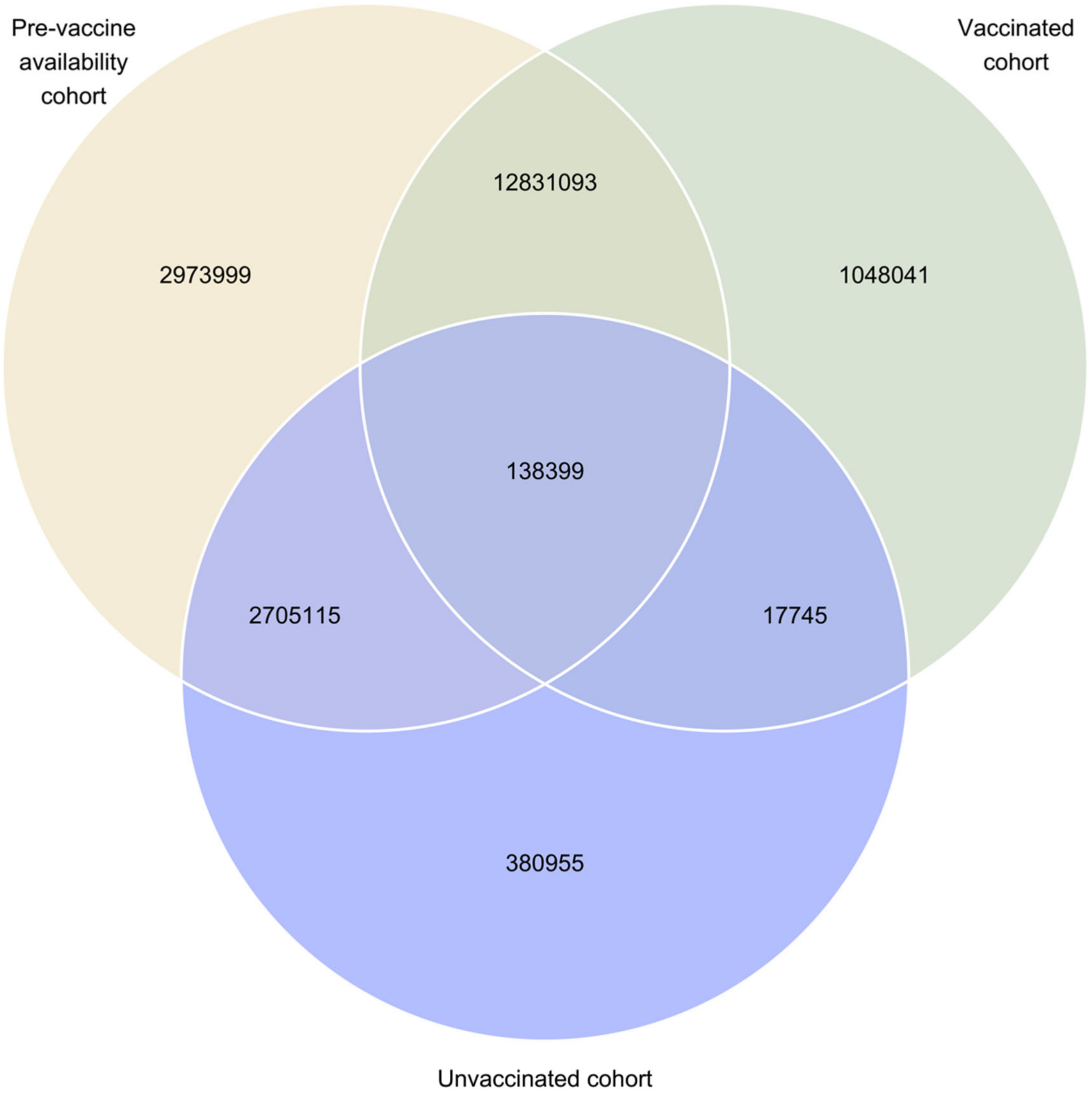

eFigure 4: Maximally and minimally adjusted hazard ratios and 95% CIs for mental illness events following diagnosis of COVID-19 in the pre-vaccine availability, vaccinated and unvaccinated cohorts. Events on the day of COVID-19 diagnosis (day 0) were excluded.

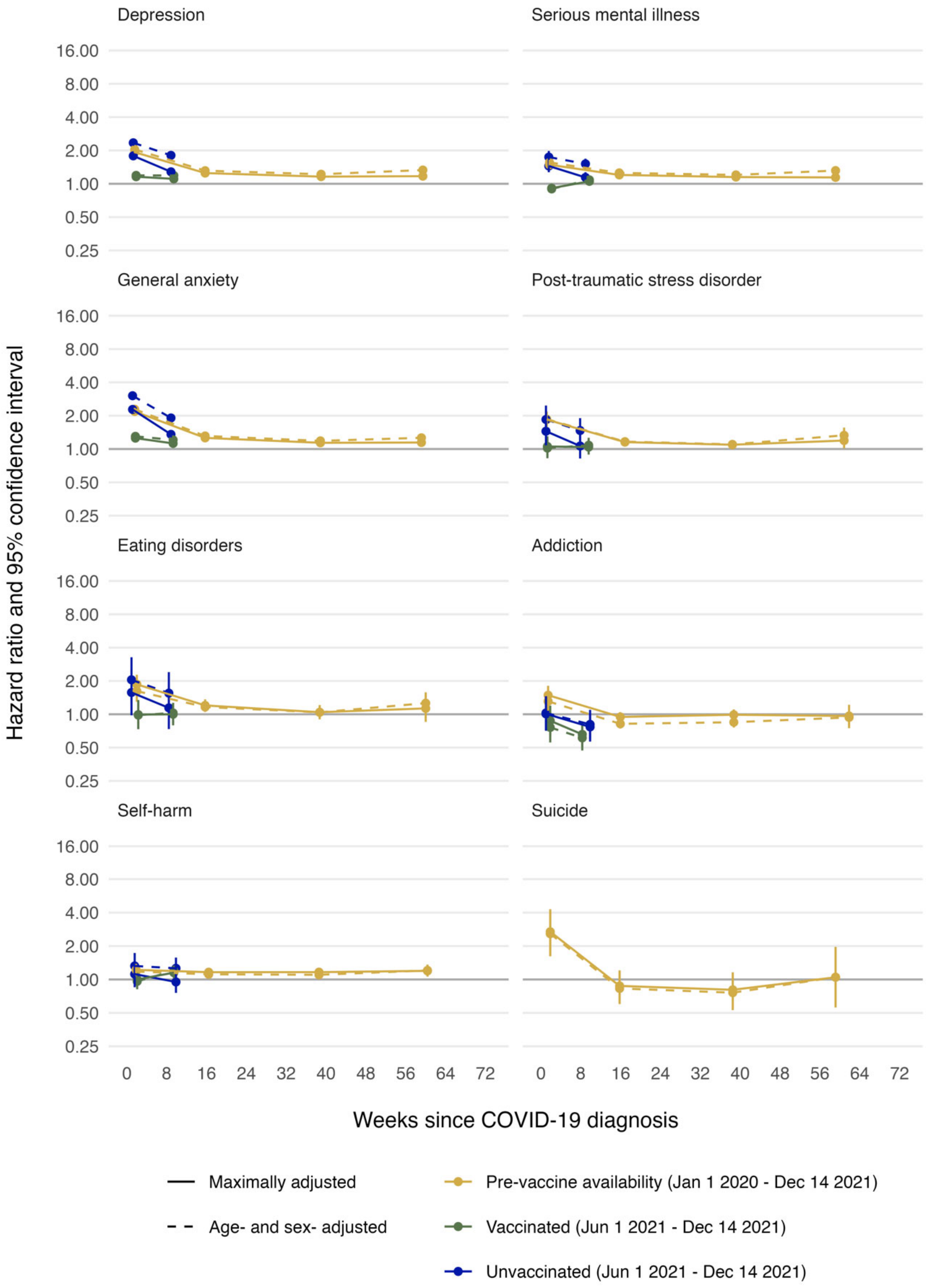

eFigure 5: Maximally adjusted hazard ratios and 95% CIs for depression and serious mental illness following diagnosis of COVID-19 in the vaccinated and unvaccinated cohorts, by history of COVID-19. Events on the day of COVID-19 diagnosis (day 0) were excluded.

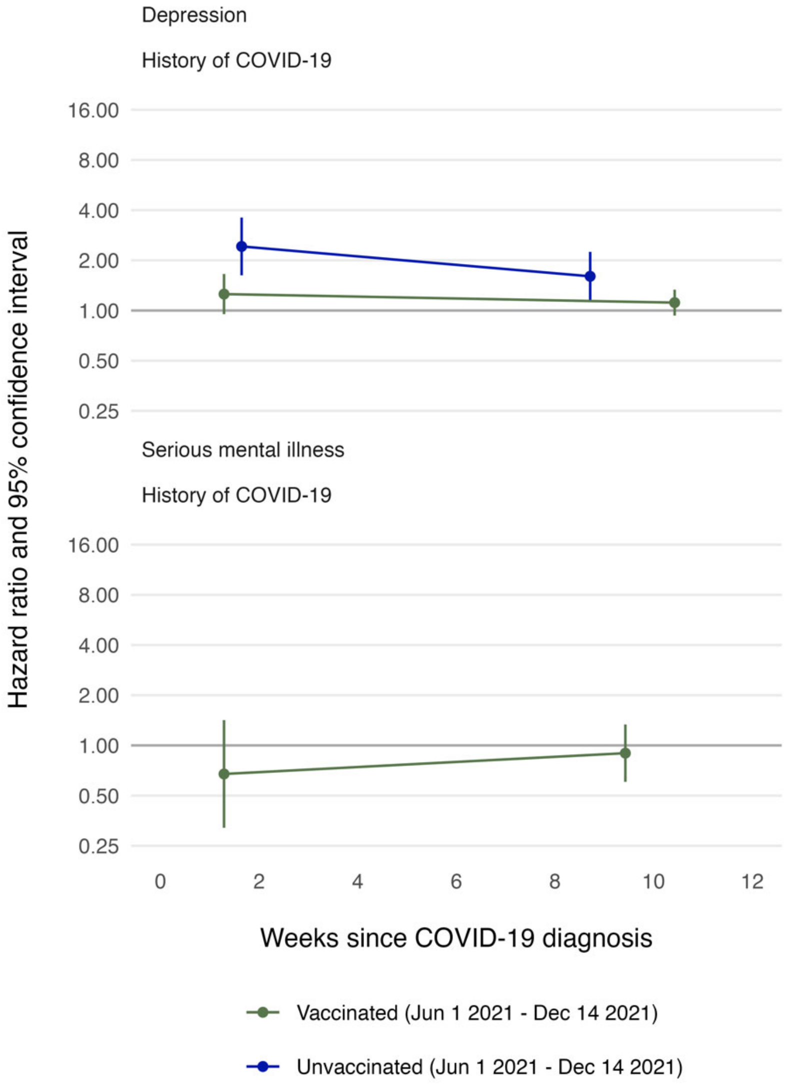

Note: there were less than 50 serious mental illness events post-COVID-19 in the unvaccinated cohort and so the model was not run.

eFigure 6: Maximally adjusted hazard ratios and 95% CIs for depression and serious mental illness following diagnosis of COVID-19 in the pre-vaccine availability, vaccinated and unvaccinated cohorts, by age group. Events on the day of COVID-19 diagnosis (day 0) were excluded.

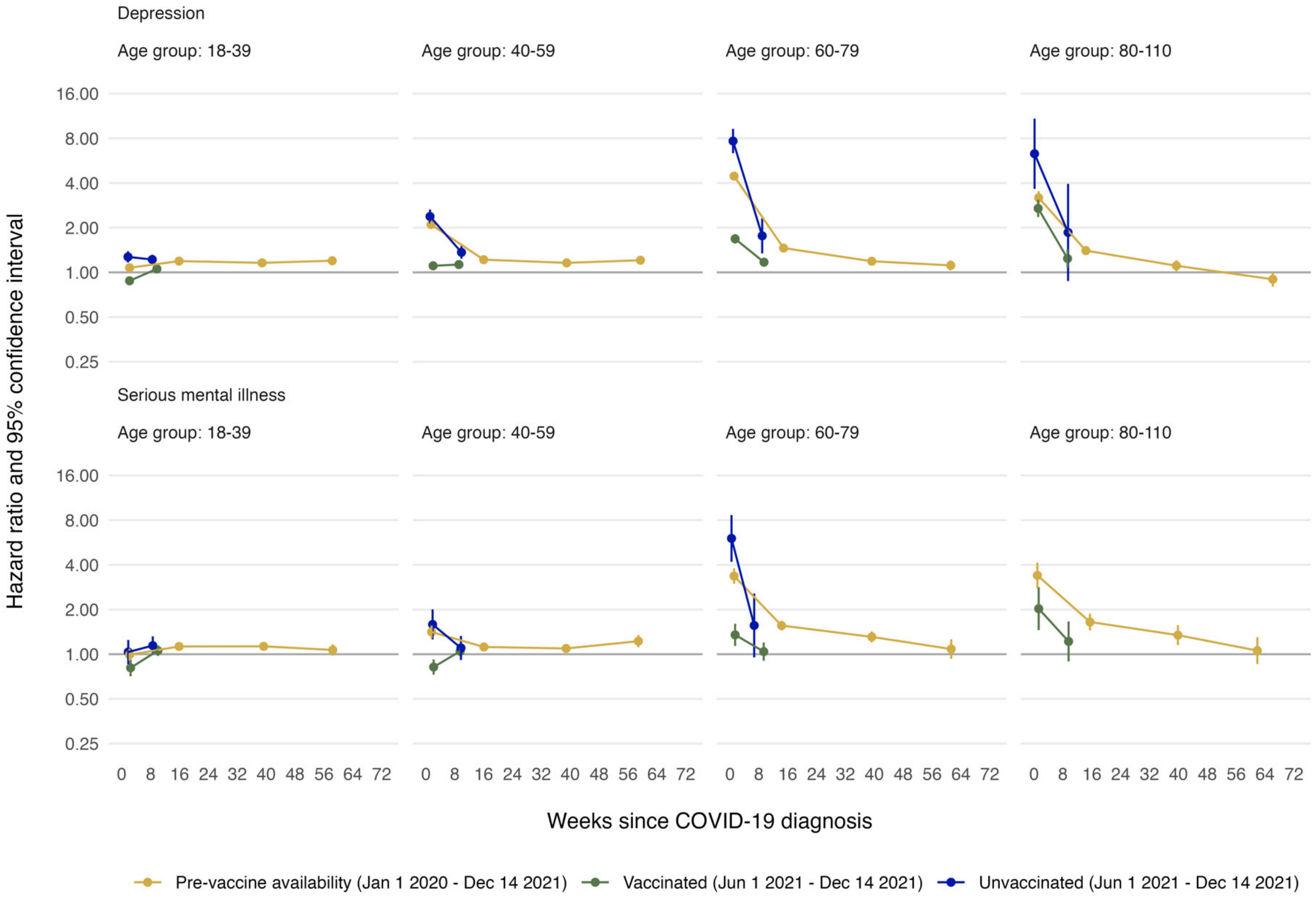

Note: There were less than 50 serious mental illness events post-COVID-19 among individuals aged 80-110 in the unvaccinated cohort and so the model was not run.

eFigure 7: Maximally adjusted hazard ratios and 95% CIs for depression and serious mental illness following diagnosis of COVID-19 in the pre-vaccine availability, vaccinated and unvaccinated cohorts, by sex. Events on the day of COVID-19 diagnosis (day 0) were excluded.

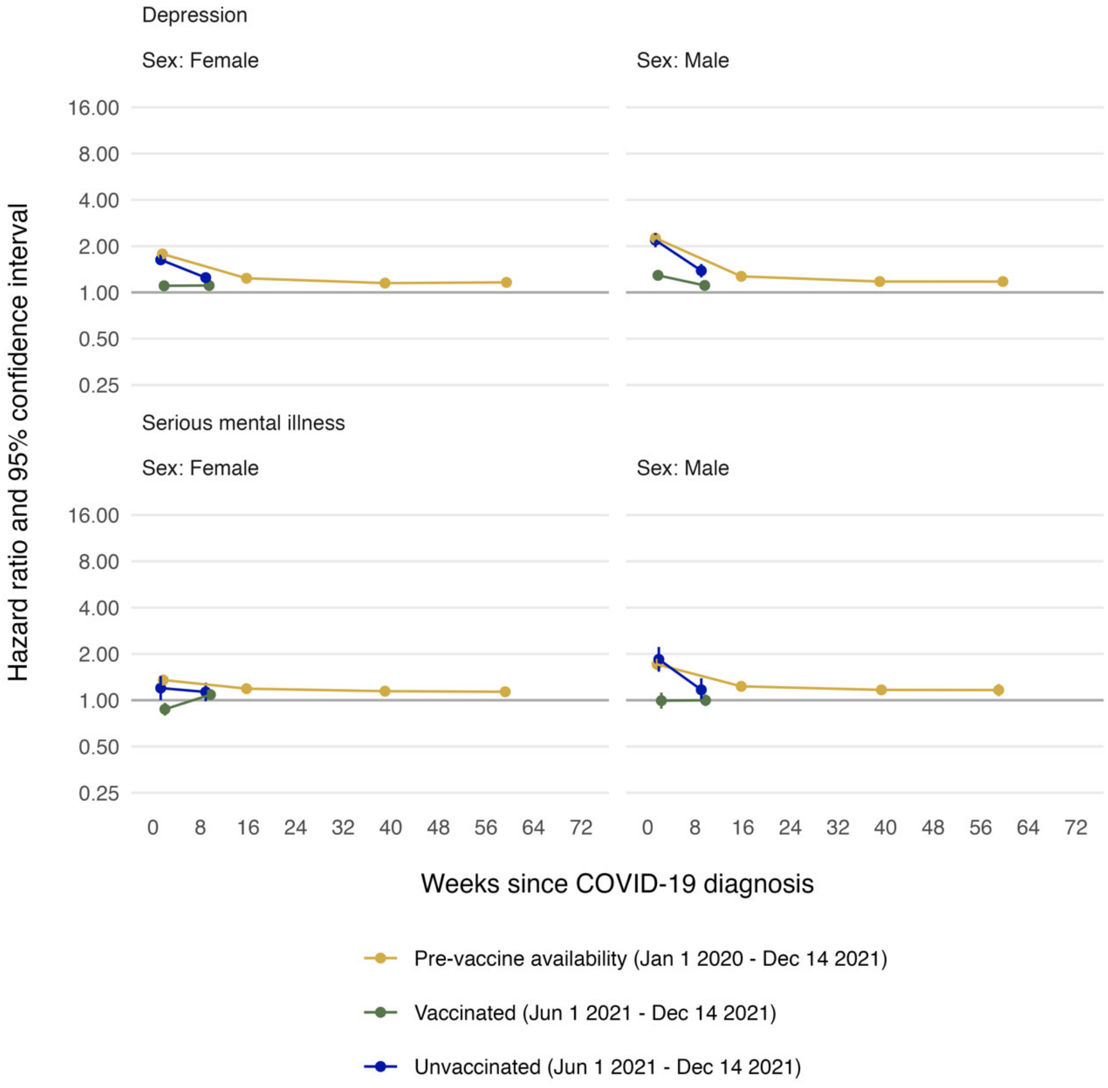

eFigure 8: Maximally adjusted hazard ratios and 95% CIs for depression and serious mental illness following diagnosis of COVID-19 in the pre-vaccine availability, vaccinated and unvaccinated cohorts, by ethnicity. Events on the day of COVID-19 diagnosis (day 0) were excluded.

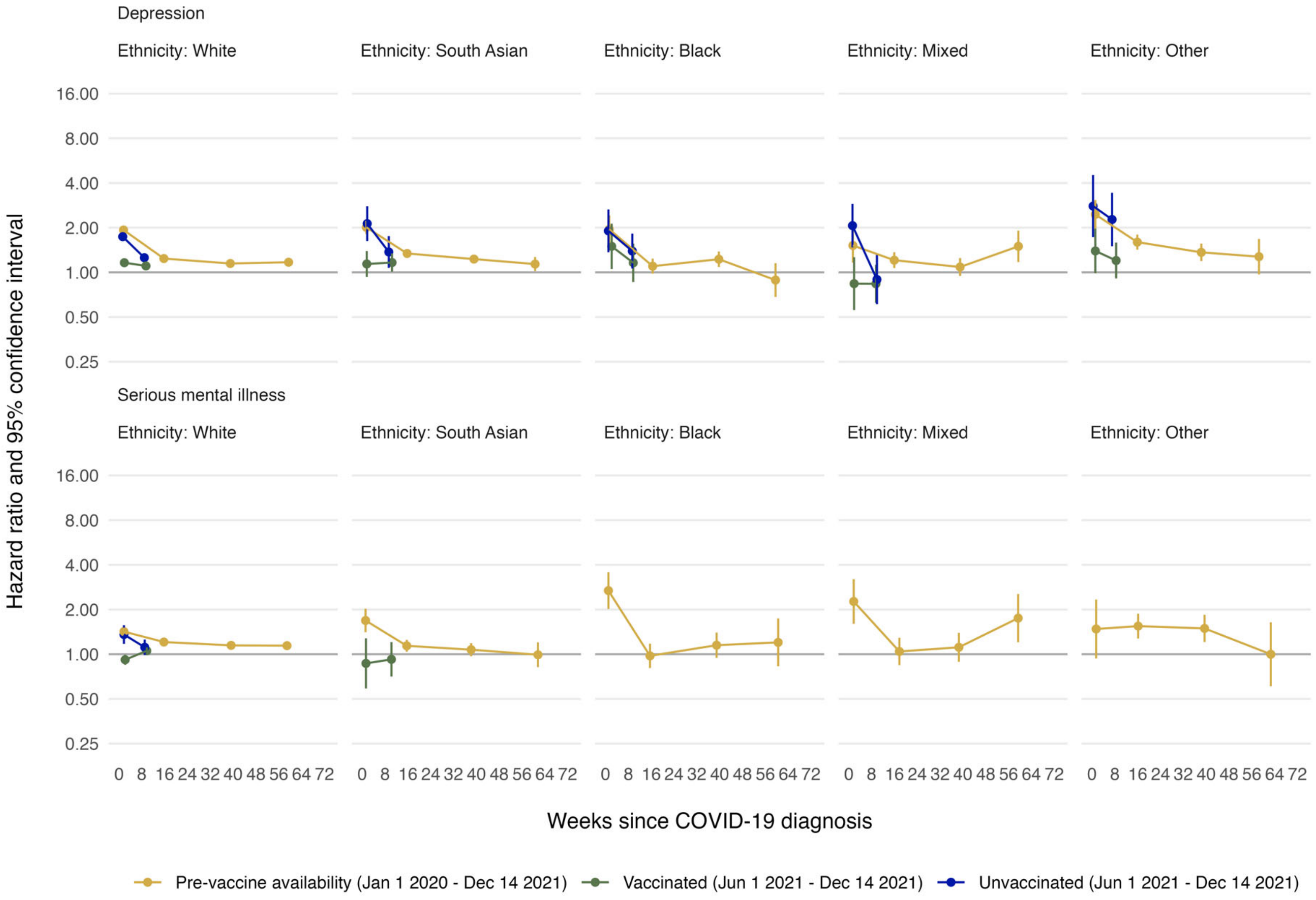

Note: There were less than 50 serious mental illness events post-COVID-19 among individuals of Black, Mixed, and Other ethnicities in the vaccinated and unvaccinated cohorts so the model was not run. There were also less than 50 serious mental illness events post-COVID-19 among individuals of South Asian ethnicity in the unvaccinated cohorts so the model was not run.

eFigure 9: Absolute excess risk up to 28 weeks for depression and serious mental illness following diagnosis of COVID-19 in the pre-vaccine availability, vaccinated and unvaccinated cohorts.

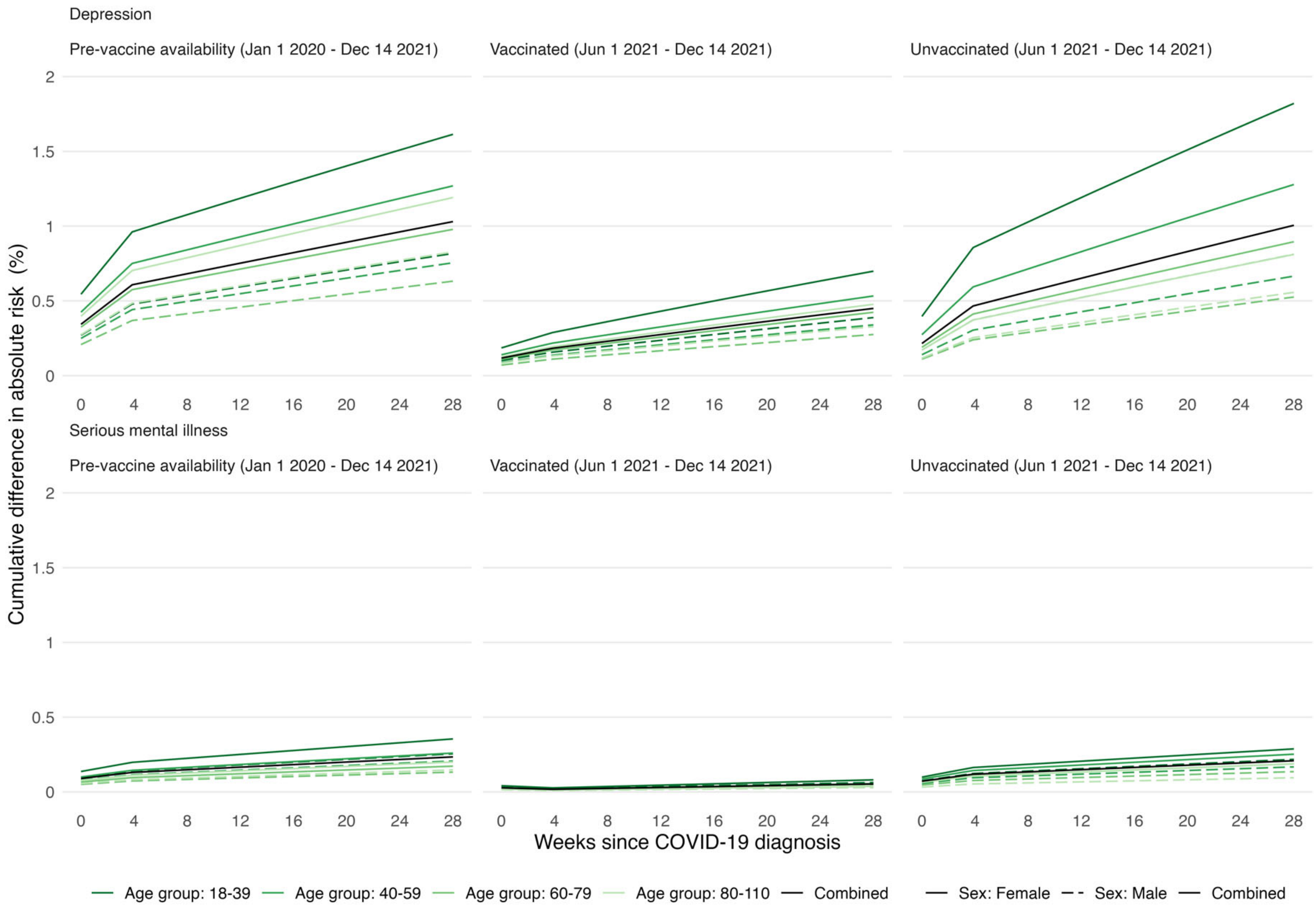

Supplement: Supplement 1. — eMethods eTable 1. Summary of covariate definitions eTable 2. Summary of cohort characteristics eTable 3. Medical history of people in the pre-vaccine availability, vaccinated and unvaccinated cohorts eTable 4. Mental illness events following diagnosis of COVID-19 in the pre-vaccine availability, vaccinated and unvaccinated cohorts, by prior history of the outcome eTable 5. Maximally adjusted hazard ratios and 95% CIs for mental illness events following diagnosis of COVID-19 in the pre-vaccine availability, vaccinated and unvaccinated cohorts eTable 6. Maximally adjusted hazard ratios and 95% CIs for mental illness events following hospitalised COVID-19 in the pre-vaccine availability, vaccinated and unvaccinated cohorts eTable 7. Maximally adjusted hazard ratios and 95% Cis for mental illness events following non-hospitalised COVID-19 in the pre-vaccine availability, vaccinated and unvaccinated cohorts eTable 8. Maximally adjusted hazard ratios and 95% CIs for mental illness events following diagnosis of COVID-19 in the pre-vaccine availability, vaccinated and unvaccinated cohorts, in people with no prior history of the outcome eTable 9. Maximally adjusted hazard ratios and 95% CIs for mental illness events following diagnosis of COVID-19 in the pre-vaccine availability, vaccinated and unvaccinated cohorts, in people with prior history of the outcome, more than six months ago eTable 10. Maximally adjusted hazard ratios and 95% CIs for mental illness events following diagnosis of COVID-19 in the pre-vaccine availability, vaccinated and unvaccinated cohorts, in people with prior history of the outcome, within six months eTable 11. Maximally adjusted hazard ratios and 95% CIs for mental illness events following diagnosis of COVID-19 in the pre-vaccine availability, vaccinated and unvaccinated cohorts, in people with history of COVID-19 eTable 12. Maximally adjusted hazard ratios and 95% CIs for mental illness events following diagnosis of COVID-19 in the pre-vaccine a [file jamapsychiatry-e242339-s001.pdf]
