# Supplementary material for: COVID-19 and Mental Illnesses in Vaccinated and Unvaccinated People
Source: JAMA Psychiatry. 2024 Aug 21;81(11):1071–80. doi: 10.1001/jamapsychiatry.2024.2339 (PMC11339697; doi:10.1001/jamapsychiatry.2024.2339)
Supplement: Supplement 2. — Members of the Longitudinal Health and Wellbeing COVID-19 National Core Study [file jamapsychiatry-e242339-s002.pdf]

Supplemental Online Content: Nonauthor Collaborators
*First name, last name, and suffix (if applicable) are required and will appear in PubMed.

*Group Name(s): Longitudinal Health and Wellbeing COVID-19 National Core Study							
*First Name and Middle Initial(s)	*Last Name	*Suffix (eg, Jr, III)	Academic Degrees	Institution	Location (city, state/province, country)	Role or Contribution, eg, chair, principal investigator	Group (if more than 1 Group listed in the byline) and/or Subgroup (eg, Steering Committee)
Agnieszka	Lemanska						
Alex	Kwong						
Alexia	Sampri						
Alicja	Rapala						
Alisia	Carnemolla						
Alun	Hughes						
Amelia	Green						
Amos	Folarin						
Amy	Roberts						
Ana	Goncalves Soares						
Andrew	Steptoe						
Andrew	Wong						
Andy	Boyd						
Andy	Gibson						
Anika	Knueppel						
Anoop	Shah						
Archie	Campbell						
Arun	Kanagaratnam						
Arun	Karthikeyan Suseeladevi						
Bang	Zheng						
Betty	Raman						
Bo	Hou						
Bozena	Wielgoszewska						
Brain	Mackenna						
Callum	Stewart						
Chao	Fang						
Charis	Bridger-Staatz						

Supplemental Online Content: Nonauthor Collaborators
*First name, last name, and suffix (if applicable) are required and will appear in PubMed.

*First Name and Middle Initial(s)	*Last Name	*Suffix (eg, Jr, III)	Academic Degrees	Institution	Location (city, state/province, country)	Role or Contribution, eg, chair, principal investigator	Group (if more than 1 Group listed in the byline) and/or Subgroup (eg, Steering Committee)
Charlotte	Booth						
Chelsea	Beckford						
Chloe	Park						
Claire	Steves						
Colm	Andrews						
Daniel	McCartney						
Daniel	Kopasker						
Diane	Foster						
Dominik	Piehlmaier						
Dylan	Williams						
Edward	Parker						
Elena	Rafeti						
Elena	Lukaschuk						
Elizabeth	Tunnicliffe						
Ellena	Badrick						
Emily	Herrett						
Emma L	Turner						
Eoin	McElroy						
Fatima	Almaghrabi						
Felix	Greaves						
Fintan	McArdle						
Fiona	Glen						
Francisco	Perez-Reche						
Gareth	Griffith						
George	Ploubidis						
Gillian	Santorelli						
Giorgio	Di Gessa						
Hannah	Woodward						
Helen	Curtis						
Annie	Herbert						
Ian	Douglas						

Supplemental Online Content: Nonauthor Collaborators
*First name, last name, and suffix (if applicable) are required and will appear in PubMed.

***First Name and Middle Initial(s)**	***Last Name**	***Suffix (eg, Jr, III)**	Academic Degrees	Institution	Location (city, state/province, country)	Role or Contribution, eg, chair, principal investigator	Group (if more than 1 Group listed in the byline) and/or Subgroup (eg, Steering Committee)
Jacqui	Oakley						
James M	Wild						
Jane	Maddock						
JD	Carpentieri						
Jess	Morley						
Jingmin	Zhu						
John	Tazare						
John	Wright						
John	Kellas						
Jonathan	Kennedy						
Kate	Northstone						
Kate	Tilling						
Kate	Mansfield						
Katharine M	Evans						
Kathryn	Mansfield						
Kathryn	Willan						
Kevin	Wang						
Kirsteen C	Campbell						
Kishan	Patel						
Laura	Fox						
Laura	Sheard						
Laura C	Saunders						
Laurie	Tomlinson						
Lee	Hamill Howes						
Liam	Smeeth						
Lidia	Nigrelli						
Linda	Nab						
Lisa	Hopcroft						
Lizzie	Huntley						
Louise	Jones						
Lucy	Finnigan						

Supplemental Online Content: Nonauthor Collaborators
*First name, last name, and suffix (if applicable) are required and will appear in PubMed.

***First Name and Middle Initial(s)**	***Last Name**	***Suffix (eg, Jr, III)**	Academic Degrees	Institution	Location (city, state/province, country)	Role or Contribution, eg, chair, principal investigator	Group (if more than 1 Group listed in the byline) and/or Subgroup (eg, Steering Committee)
Lucy	Teece						
Mark	Green						
Matthew	Crane						
Maxim	Freydin						
Michael	Parker						
Michael	Green						
Milla	Kibble						
Nathan	Cheetham						
Nicholas	Timpson						
Olivia	Hamilton						
Paola	Zaninotto						
Paz	Garcia						
Peter	Jezzard						
Rebecca	Whitehorn						
Rebecca	Rhead						
Renin	Toms						
Richard	Thomas						
Richard	Silverwood						
Richard	Dobson						
Robert	Willans						
Robin	Flaig						
Rosie	McEachan						
Ruth	Mitchell						
Ruth	Bowyer						
Ruth	Costello						
Sam	Parsons						
Samantha	Berman						
Samantha	Ip						
Sarah	Baz						
Scott	Walker						
Shah	Syed Ahmar						

Supplemental Online Content: Nonauthor Collaborators
*First name, last name, and suffix (if applicable) are required and will appear in PubMed.

***First Name and Middle Initial(s)**	***Last Name**	***Suffix (eg, Jr, III)**	Academic Degrees	Institution	Location (city, state/province, country)	Role or Contribution, eg, chair, principal investigator	Group (if more than 1 Group listed in the byline) and/or Subgroup (eg, Steering Committee)
Richard	Shaw						
Sheikh	Aziz						
Sinead	Langan						
Sinead	Brophy						
Spiros	Denaxas						
Stefan	Neubauer						
Stefan	Piechnik						
Stela	McLachlan						
Stephen	Smith						
Steve	Sharp						
Teri	North						
Theocharis	Kromydas						
Thomas	Cowling						
Tiffany	Yang						
Tom	Bolton						
Vanessa	Ferreira						
Vittal	Katikireddi						
Viyaasan	Mahalingasivam						
Wels	Jacques						
William	Hulme						
Yatharth	Ranjan						
Zeena-Britt	Sanders						
