# Supplementary material for: COVID-19 and Mental Illnesses in Vaccinated and Unvaccinated People
Source: JAMA Psychiatry. 2024 Aug 21;81(11):1071–80. doi: 10.1001/jamapsychiatry.2024.2339 (PMC11339697; doi:10.1001/jamapsychiatry.2024.2339)
Supplement: Supplement 3. — Data sharing statement [file jamapsychiatry-e242339-s003.pdf]

Data Sharing Statement

Walker. COVID-19 and Mental Illnesses in Vaccinated and Unvaccinated People. *JAMA Psychiatry*. Published August 21, 2024. doi:10.1001/jamapsychiatry.2024.2339

Data
Data available: No

Additional Information
Explanation for why data not available: All data were linked, stored and analysed securely within the OpenSAFELY platform: https://www.opensafely.org/. Data include pseudonymised data such as coded diagnoses, medications and physiological parameters. No free text data are included. All code and code lists are shared openly for review and re-use under an MIT open license (https://github.com/opensafely/post-covid-mentalhealth). Detailed pseudonymised patient data is potentially re-identifiable and therefore not shared.
